# Promotion of Cx26 mutants located in TM4 region for membrane translocation successfully rescued hearing loss

**DOI:** 10.7150/thno.112225

**Published:** 2025-04-22

**Authors:** Yan-Jun Zong, Xiao-Zhou Liu, Xin-Yu Shi, Zheng-Dong Zhao, Yu Sun

**Affiliations:** 1 Department of Otorhinolaryngology, Union Hospital, Tongji Medical College, Huazhong University of Science and Technology, Wuhan 430022, China.; 2 Institute of Otorhinolaryngology, Union Hospital, Tongji Medical College, Huazhong University of Science and Technology, Wuhan 430022, China.; 3 Hubei Province Clinic Research Center for Deafness and Vertigo, Wuhan 430022, China.

**Keywords:** Cx26, hearing loss, mutants, SPTBN1, treatment, cytoskeleton

## Abstract

**Rationale:** The *GJB2* gene, which encodes connexin 26 (Cx26), is recognized as the leading cause of non-syndromic hereditary hearing loss. In clinical settings, a total of 131 Cx26 mutations have been identified in association with hearing loss. Certain Cx26 mutants display normal structural and functional properties but fail to translocate to the plasma membrane. Enhancing the membrane localization of these mutants may provide a promising strategy for rescuing hearing loss and hair cell degeneration.

**Methods:** This study investigated the membrane localization of Cx26 using *in vitro* cell lines, cultured cochlear explants, and *in vivo* murine models. Key proteins involved in the membrane localization of Cx26 were identified and validated through immunoprecipitation-mass spectrometry (IP-MS) and co-immunoprecipitation (Co-IP). Additionally, cell lines and murine models harboring Cx26 mutants were developed to evaluate the effects of Narciclasine on enhancing the membrane localization of these mutants, as well as its potential to rescue hearing loss.

**Results:** The membrane localization of Cx26 was dependent on the integrity of the intracellular transport network consisting of microtubules, actin microfilaments, and the Golgi apparatus. Additionally, SPTBN1 played a significant role in this process. The transmembrane domain 4 (TM4) region exhibited a strong association with the membrane localization of Cx26, and Cx26 mutants located in TM4 region retained in the cytoplasm. Narciclasine promoted cytoskeletal development, thereby enhancing the membrane localization of Cx26 mutants retained in the cytoplasm. This process helped to reconstruct the inner ear gap junction network and rescue hearing loss and hair cell degeneration.

**Conclusion:** These findings present that enhancing the membrane localization of Cx26 mutants can significantly improve auditory function. This strategy offers a potential therapeutic approach for addressing hereditary sensorineural hearing loss associated with *GJB2* mutations.

## Introduction

*GJB2* mutations are the leading cause of non-syndromic hereditary hearing loss, accounting for approximately 18% 1, 2. To date, over 130 distinct *GJB2* mutations associated with hearing loss have been identified in clinical settings. The p.Leu79del (c.235delC) variant is the most prevalent among Asian populations 3, while the c.35delG mutation is recognized as the primary hotspot in Caucasian individuals 4-6, and the c.167delT mutation is the most common in the Ashkenazi Jewish population 7. Despite the identification of these mutations, there are currently no effective interventions targeting the underlying pathogenesis of *GJB2*-associated hereditary hearing loss 8-16. Treatment options primarily consist of hearing aids and cochlear implants 17. However, the effectiveness of these interventions remains unsatisfactory 18-24.

The *GJB2* gene encodes connexin 26 (Cx26), which is the most prevalent connexin in the cochlea 25-32. Cx26 facilitates the bidirectional transport of intercellular cytoplasmic ions, second messengers, and other substances with molecular weights less than 1.5 kDa, such as inositol triphosphates (IP3) 33-40. Previous studies utilizing cell models derived from population mutants have demonstrated that certain Cx26 mutants (Mut-Cx26s) exhibit deficiencies in membrane localization, leading to their retention in various intracellular structures, thereby preventing their localization to the plasma membrane for channel functions. For example, the Mut-Cx26 p.Gly12Arg, associated with keratitis-ichthyosis-deafness (KID) syndrome, is retained in the perinuclear region 41 and fails to reach cell-cell junctions. Furthermore, immunofluorescence studies reveal that the Mut-Cx26 p.Arg184Pro colocalizes with the Golgi resident protein GM130, confirming its retention within the Golgi apparatus 42.

Notably, many point or truncation mutations of Cx26 exhibit normal structural and functional characteristics but fail to translocate to the plasma membrane. This observation suggests that providing the complete Cx26 protein may not be essential. Instead, enhancing the re-transport of these mutants, which encounter barriers to membrane localization, to the plasma membrane could potentially restore their function and rescue hearing loss. Therefore, investigating the Cx26 membrane transport pathway and identifying strategies to enhance the membrane localization of Cx26 mutants is of vital importance. In this study, we conducted a systematic exploration of the membrane localization of Cx26 and the key proteins involved in this process. We also developed Mut-Cx26 cell lines *in vitro* and created Mut-Cx26 murine models. Additionally, we used Narciclasine to promote cytoskeleton development, thereby enhancing the membrane localization of Mut-Cx26s retained in the cytoplasm, with the aim of rescuing hearing loss and hair cell degeneration.

## Results

### Dependence of Cx26 plasma membrane localization on intact intracellular transport network

#### Reduced Cx26 on the plasma membrane after microtubules disruption

**Figure 1:**
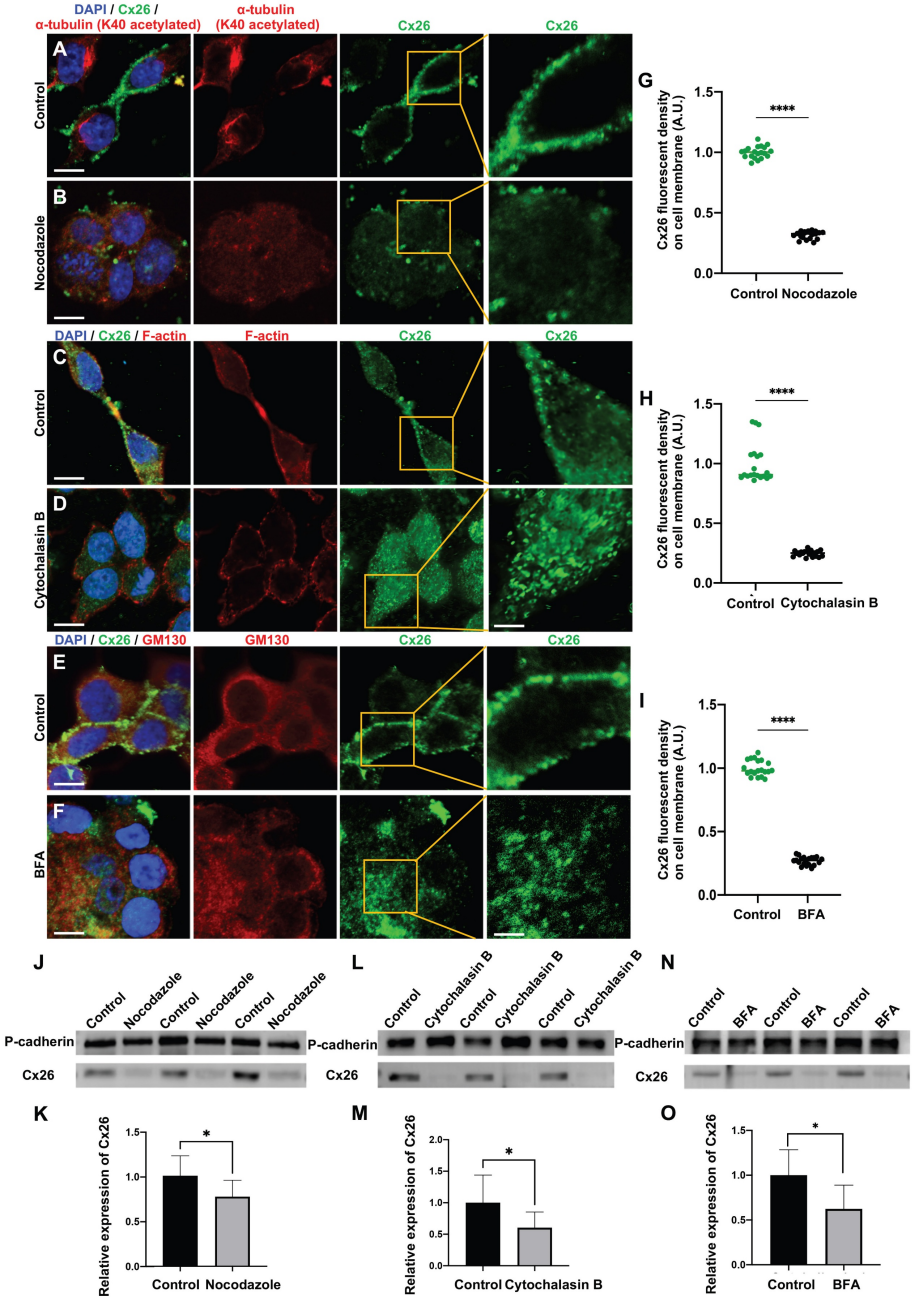
** The normal membrane localization of Cx26 is dependent on microtubules, actin microfilaments, and the Golgi apparatus *in vitro*. (A-B)** Immunofluorescent staining of Cx26 (green) and α-tubulin (K40 acetylated) (red) in the control group (A) and in the nocodazole-treated group (B) *in vitro*. **(C-D)** Immunofluorescent staining of Cx26 (green) and F-actin (red) in the control group (C) and in the Cytochalasin B-treated group (D) *in vitro*.** (E-F)** Immunofluorescent staining of Cx26 (green) and GM130 (red) in the control group (E) and in the BFA-treated group (F) *in vitro*. **(G-I)** Quantification of the Cx26 fluorescent density on plasma membrane from the control group and the treatment group *in vitro*. **(J-K)** Western blot and histogram showing Cx26 protein levels on the plasma membrane in the control group and in the nocodazole-treated group *in vitro*. **(L-M)** Western blot and histogram showing Cx26 protein levels on the plasma membrane in the control group and in the Cytochalasin B-treated group *in vitro*. **(N-O)** Western blot and histogram showing Cx26 protein levels on the plasma membrane in the control group and in the BFA-treated group *in vitro*. Scale bars: 10 μm (panels A-F), and 3 μm (partial enlargement in panels A-F). *P < 0.05, **P < 0.005, ***P < 0.001, ****P < 0.0001.

**Figure 2:**
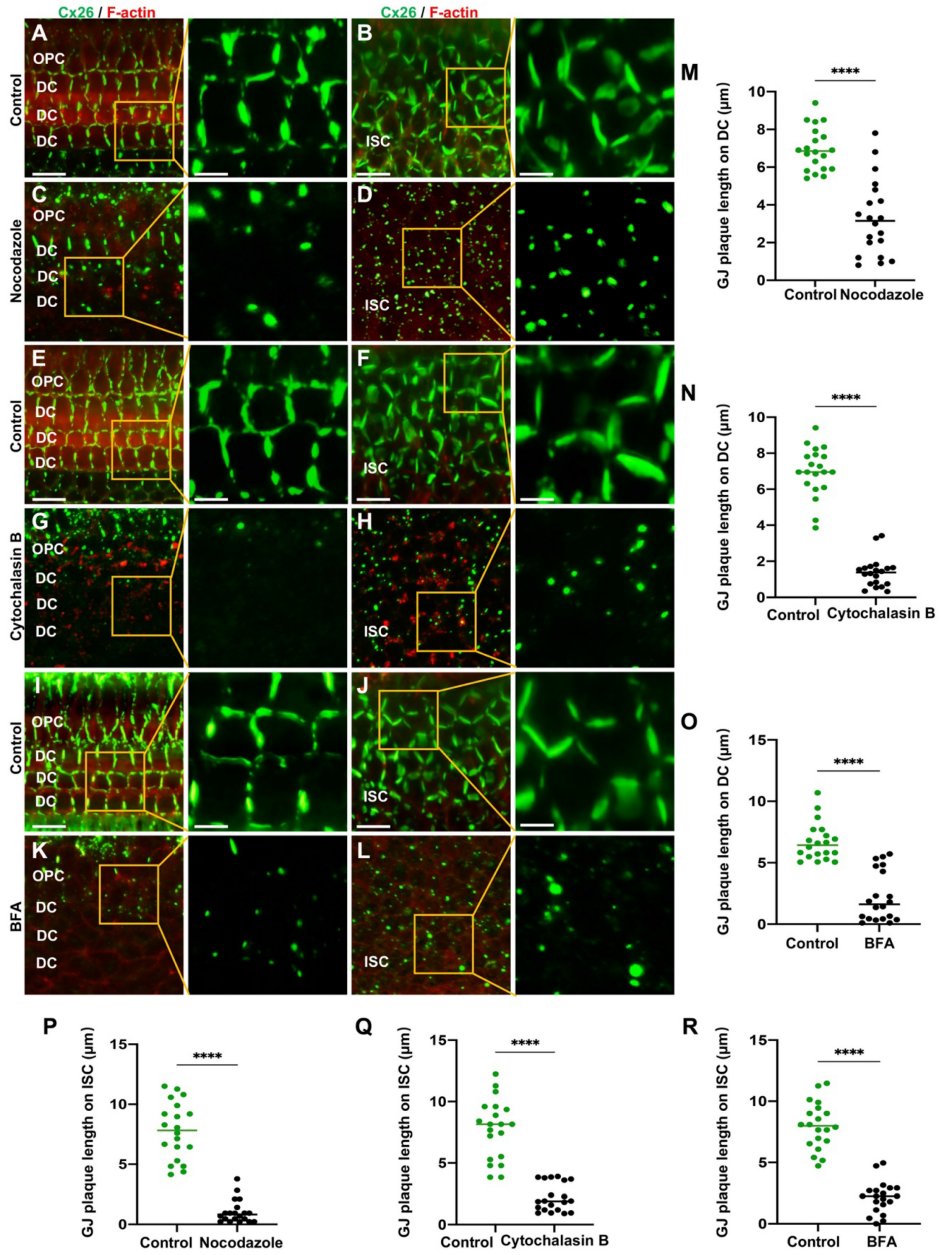
** The normal plasma membrane localization of Cx26 is dependent on microtubules, actin microfilaments, and the Golgi apparatus in the cochlear explants. (A-D)** Immunofluorescent staining of Cx26 (green) and F-actin (red) in the control group (A-B) and in the nocodazole-treated group (C-D) in DCs (A, C) and ISCs (B, D) in the cochlear explants. **(E-H)** Immunofluorescent staining of Cx26 (green) and F-actin (red) in the control group (E-F) and in the Cytochalasin B-treated group (G-H) in DCs (E, G) and ISCs (F, H) in the cochlear explants. **(I-L)** Immunofluorescent staining of Cx26 (green) and F-actin (red) in the control group (I-J) and in the BFA-treated group (K-L) in DCs (I, K) and ISCs (J, L) in the cochlear explants. **(M-R)** Quantification of the length of GJPs on DCs (M-O) and ISCs (P-R) from the control group and the treatment group in the cochlear explants. Scale bars: 20 μm (panels A-L), and 7 μm (partial enlargement in panels A-L). *P < 0.05, **P < 0.005, ***P < 0.001, ****P < 0.0001.

**Figure 3:**
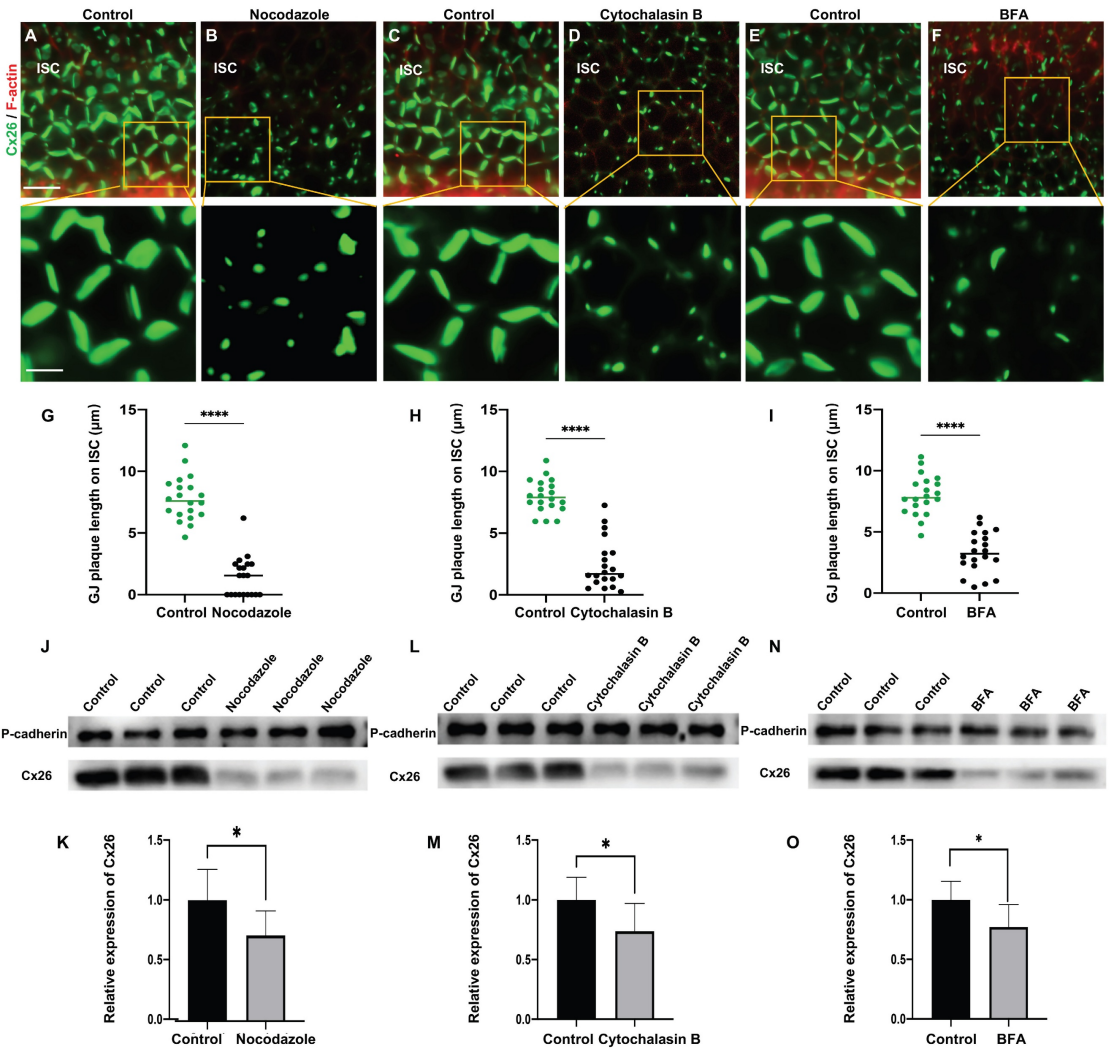
** The normal plasma membrane localization of Cx26 is dependent on microtubules, actin microfilaments, and the Golgi apparatus *in vivo*. (A**-**B)** Immunofluorescent staining of Cx26 (green) and F-actin (red) in the control group (A) and in the nocodazole-treated group (B) in ISCs *in vivo*.** (C**-**D)** Immunofluorescent staining of Cx26 (green) and F-actin (red) in the control group (C) and in the Cytochalasin B-treated group (D) in ISCs *in vivo*. **(E**-**F)** Immunofluorescent staining of Cx26 (green) and F-actin (red) in the control group (E) and in the BFA-treated group (F) in ISCs *in vivo*. **(G-I)** Quantification of the length of GJPs on ISCs from the control group and the treatment group *in vivo*.** (J-O)** Western blot and histogram showing Cx26 protein levels on the plasma membrane in the control group and in the treatment group. Scale bars: 20 μm (panels A-F), and 7 μm (partial enlargement in panels A-F). *P < 0.05, **P < 0.005, ***P < 0.001, ****P < 0.0001.

The connexin secretion pathway is posited to involve three primary components, namely microtubules, actin microfilaments, and the Golgi apparatus 43-46. This study investigated the impact of various specific inhibitors on these organelles and the membrane localization of Cx26 at three distinct levels: *in vitro* cell lines overexpressing wild type Cx26 (WT-Cx26) (Figure 1A-O), cultured cochlear explants (Figure 2A-R), and *in vivo* murine models (Figure 3A-O). Nocodazole, a β-tubulin binding agent, was used to interfere with microtubule assembly and disassembly 47. The objective was to determine the dependence of Cx26 membrane localization upon the integrity of microtubules. Nocodazole treatment resulted in complete microtubule depolymerization within 5 h, as evidenced by α-tubulin (K40 acetylated) staining (Figure 1A-B). This confirmed a substantial disruption of microtubule structure. In the control group, WT-Cx26 was targeted to the plasma membrane and assembled into large gap junction patches (GJPs) (Figure 1A). However, following 5 h of nocodazole treatment, WT-Cx26 displayed a diffuse cytoplasmic localization (Figure 1B), accompanied by a remarkable decrement of approximately 68% in Cx26 fluorescent density on plasma membrane (Figure 1G).

In cultured cochlear explants, Cx26 GJPs formed linear structures along the adjacent membranes of Deiters' cells (DCs) (Figure 2A) and inner sulcus cells (ISCs) (Figure 2B). Upon nocodazole treatment, the length of GJPs on DCs and ISCs reduced 52.7% (Figure 2C) and 86.5% (Figure 2D), respectively. This indicated significantly impaired transport of Cx26 to the plasma membrane. Additionally, the length of GJPs on ISC membranes was reduced by approximately 81.7% following microtubule disruption *in vivo* (Figure 3A-B). Collectively, these findings suggested reduced Cx26 on the plasma membrane upon the disruption of microtubules, thereby establishing the dependence of normal Cx26 membrane localization on microtubule integrity. Western blot analysis quantitatively corroborated a significant decrease in the amount of Cx26 on the plasma membrane following microtubule disruption (Figure 1J-K, Figure 3J-K), with no change in total cellular Cx26 levels.

#### Reduced Cx26 on the plasma membrane after actin microfilaments disruption

Cytochalasin B is known to bind to the barbed ends of actin microfilaments, thereby inhibiting actin polymerization. Herein, in order to examine the reliance of the normal membrane localization of Cx26 on actin microfilaments, Cytochalasin B was used to disrupt actin microfilaments. As illustrated in Figure 1C, the control group exhibited a characteristic filamentous arrangement of actin microfilaments. However, following the application of Cytochalasin B, a significant reduction in these fibrous structures and some instances of complete depolymerization were observed (Figure 1D), as confirmed by FITC-phalloidin staining.

Furthermore, the Cx26 fluorescent density on plasma membrane was reduced by 75.2% compared with the control group (Figure 1H) *in vitro*. In cochlear explants, the presence of actin aggregates dispersed throughout the cytoplasm suggested that Cytochalasin B effectively disrupted the actin microfilaments (Figure 2E-H). This disruption significantly reduced Cx26 gap junction formation, decreasing the length of GJPs by approximately 80.3% for DCs (Figure 2N) and 71.6% for ISCs (Figure 2Q). Consistent results were observed in a WT murine model (Figure 3C-D), where the GJPs length on ISCs was reduced by 68.9% (Figure 3H). Collectively, these findings indicated that the normal membrane localization of Cx26 was contingent upon the structural integrity of actin microfilaments. Additionally, western blot analyses corroborated these conclusions in both cellular contexts (Figure 1L-M) and cochlear explants (Figure 3L-M).

#### Reduced Cx26 on the plasma membrane after the Golgi apparatus disruption

Brefeldin A (BFA) represents a reversible inhibitor of protein transport that disrupts the structural integrity and functional capacity of the Golgi apparatus. In this study, BFA was used to investigate the reliance of the normal membrane localization of Cx26 on the Golgi apparatus. Immunostaining with anti-GM130 demonstrated that the Golgi apparatus was disassembled in the treated group (Figure 1E-F), thereby confirming the efficacy of BFA. Notably, the Cx26 fluorescent density on plasma membrane was reduced by 73.1% (Figure 1I) *in vitro*. In the cochlear explants, the length of GJPs was reduced by 84.8% in DCs and by 73.1% in ISCs (Figure 2I-L) following BFA treatment. Additionally, the length of GJPs on ISCs (Figure 3E-F) was reduced by 58.9% *in vivo*. Furthermore, western blot analysis revealed a substantial decrease in Cx26 on the plasma membrane following the Golgi apparatus disruption (Figure 1N-O, Figure 3N-O). These findings confirmed that the normal Cx26 plasma membrane localization relied on an intact Golgi apparatus.

Cx26 membrane localization was a complex process contingent upon the integrity and function of microtubules, actin microfilaments, and the Golgi apparatus.

### SPTBN1's involvement in the membrane localization of Cx26 *in vitro* and *in vivo*

**Figure 4:**
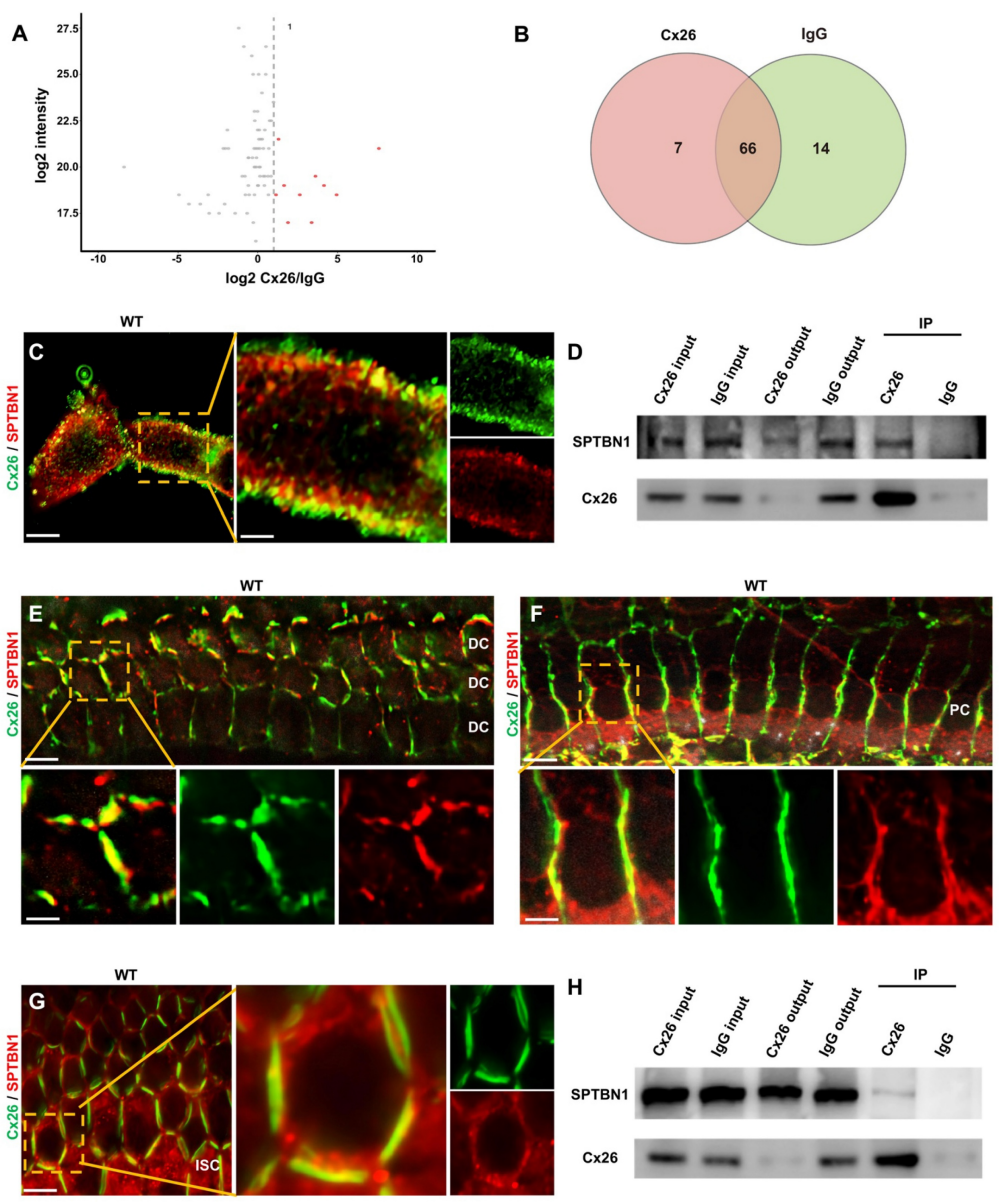
** SPTBN1 participates in the membrane localization of Cx26 *in vitro* and *in vivo*. (A)** Volcano plots highlights differentially abundant proteins shown in red identified by IP-MS from the anti-Cx26 immunoprecipitation group and the anti-IgG immunoprecipitation group. **(B)** Venn diagram of IP-MS result indicates that seven proteins may be associated with the plasma membrane localization of Cx26.** (C)** Immunofluorescent staining of Cx26 (green) and SPTBN1 (red) *in vitro*. **(D)** CO-IP results of Cx26 with SPTBN1 *in vitro*. **(E-G)** Immunofluorescent staining of Cx26 (green) and SPTBN1 (red) in DCs (E), PCs (F), and ISCs (G) *in vivo*. **(H)** CO-IP results of Cx26 with SPTBN1 *in vivo*. Scale bars: 10 μm (panel C), 3 μm (partial enlargement in panel C), 20 μm (panels E-G), and 7 μm (partial enlargement in panels E-G).

To examine the key proteins implicated in the membrane localization of Cx26, immunoprecipitation-mass spectrometry (IP-MS) was hereby performed. The findings from the IP-MS suggested a potential interaction between SPTBN1 and Cx26 (Figure 4A-B), which was subsequently validated through immunofluorescence analysis. This analysis revealed a significant co-localization of Cx26 and SPTBN1 within the membrane region *in vitro* (Figure 4C). Furthermore, co-immunoprecipitation (Co-IP) experiments further supported the interaction between these two proteins (Figure 4D). Moreover, the interaction of Cx26 and SPTBN1 was also investigated *in vivo*. The immunofluorescence results indicated significant co-localization of Cx26 with SPTBN1 in DCs (Figure 4E), pillar cells (PCs) (Figure 4F), and ISCs (Figure 4G). Additionally, Co-IP results corroborated the interaction of Cx26 with SPTBN1 *in vivo* (Figure 4H).

**Figure 5:**
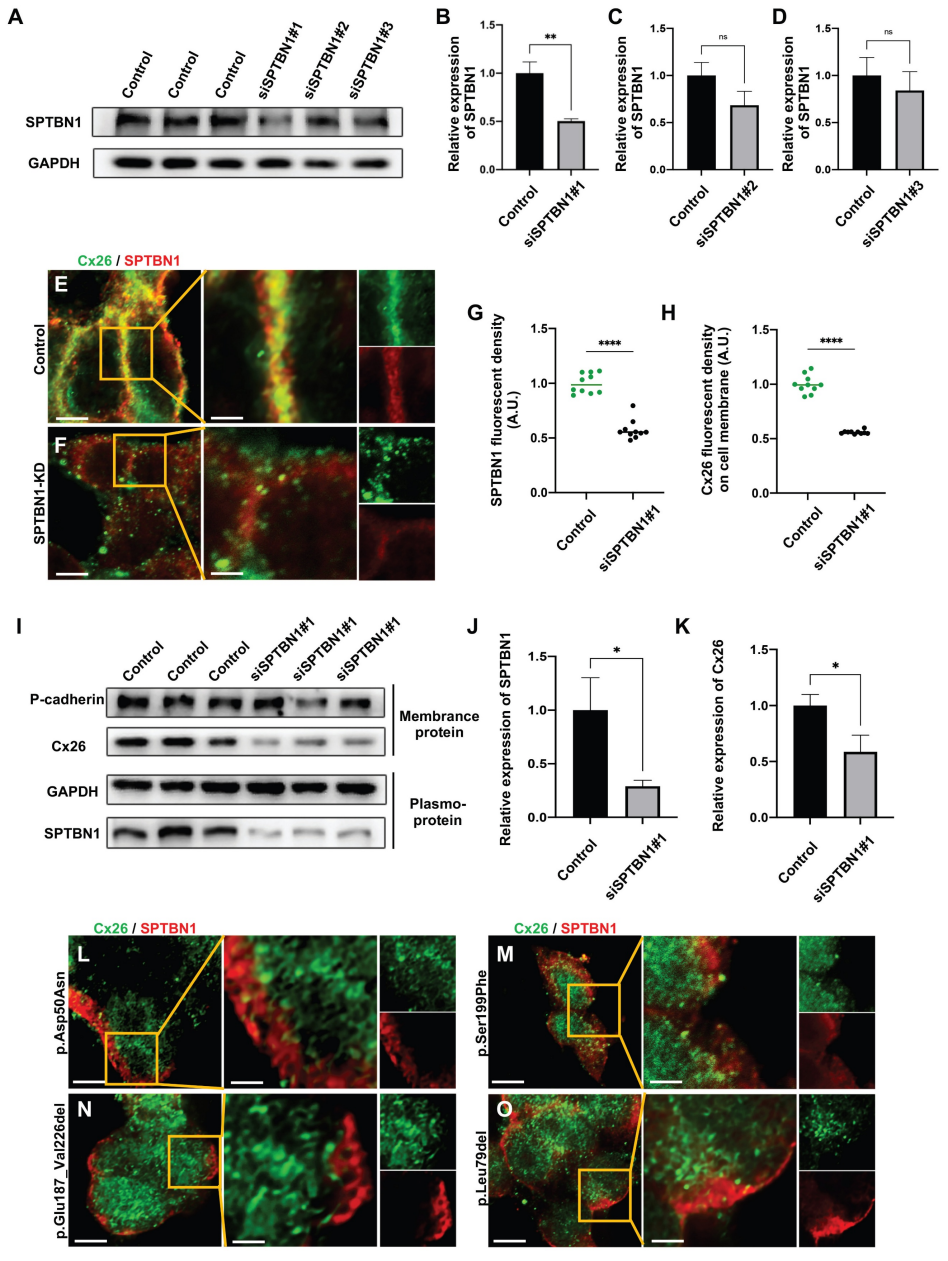
** The knockdown of SPTBN1 leads to a significant decrease in Cx26 on the plasma membrane *in vitro*. (A-D)** Western blot and histogram showing SPTBN1 protein levels in the control groups and in the SPTBN1 knockdown groups *in vitro*. **(E-F)** Immunofluorescent staining of Cx26 (green) and SPTBN1 (red) in the control group (E) and in the SPTBN1 knockdown group (F) *in vitro*. **(G-H)** Quantification of the SPTBN1 fluorescence intensity (G) and Cx26 fluorescence intensity on plasma membrane (H) from the control group and the SPTBN1 knockdown group *in vitro*.** (I-K)** Western blot and histogram showing SPTBN1 protein levels and Cx26 protein levels on the plasma membrane in the control groups and in the SPTBN1 knockdown groups *in vitro*. **(L-O)** Immunofluorescent staining of Mut-Cx26 (p.Asp50Asn (L), p.Ser199Phe (M), p.Glu187_Val226del (N), and p.Leu79del (O)) (green) and SPTBN1 (red) *in vitro*. Scale bars: 10 μm (panels E-F, panels L-O), and 3 μm (partial enlargement in panels E-F, and panels L-O). *P < 0.05, **P < 0.005, ***P < 0.001, ****P < 0.0001.

The SPTBN1 knockdown assay was used to confirm the involvement of SPTBN1 in the membrane localization of Cx26 *in vitro*. As illustrated in Figure 5A-D, the knockdown efficiency of SPTBN1 exceeded 50%. The interfering sequence exhibiting the most conspicuous knockdown efficiency was chosen for further experiments. Following the knockdown of SPTBN1, a significant reduction in Cx26 was noticed on the plasma membrane, impeding the formation of GJPs (Figure 5E-H). Quantitative analysis through western blot revealed a 41.3% decrease in Cx26 on the plasma membrane post-SPTBN1 knockdown (Figure 5I-K). Furthermore, immunofluorescence results indicated significantly decreased co-localization of Mut-Cx26 with SPTBN1 *in vitro* (Figure 5L-O), suggesting that mutations occurring in the transmembrane domain 4 (TM4) region might reduce the interaction between Cx26 and SPTBN1, thereby impairing the transport of Cx26 to the plasma membrane.

### Mut-Cx26 (p.Asp50Asn, p.Ser199Phe, p.Glu187_Val226del, and p.Leu79del) retained in the cytoplasm *in vitro*

**Figure 6:**
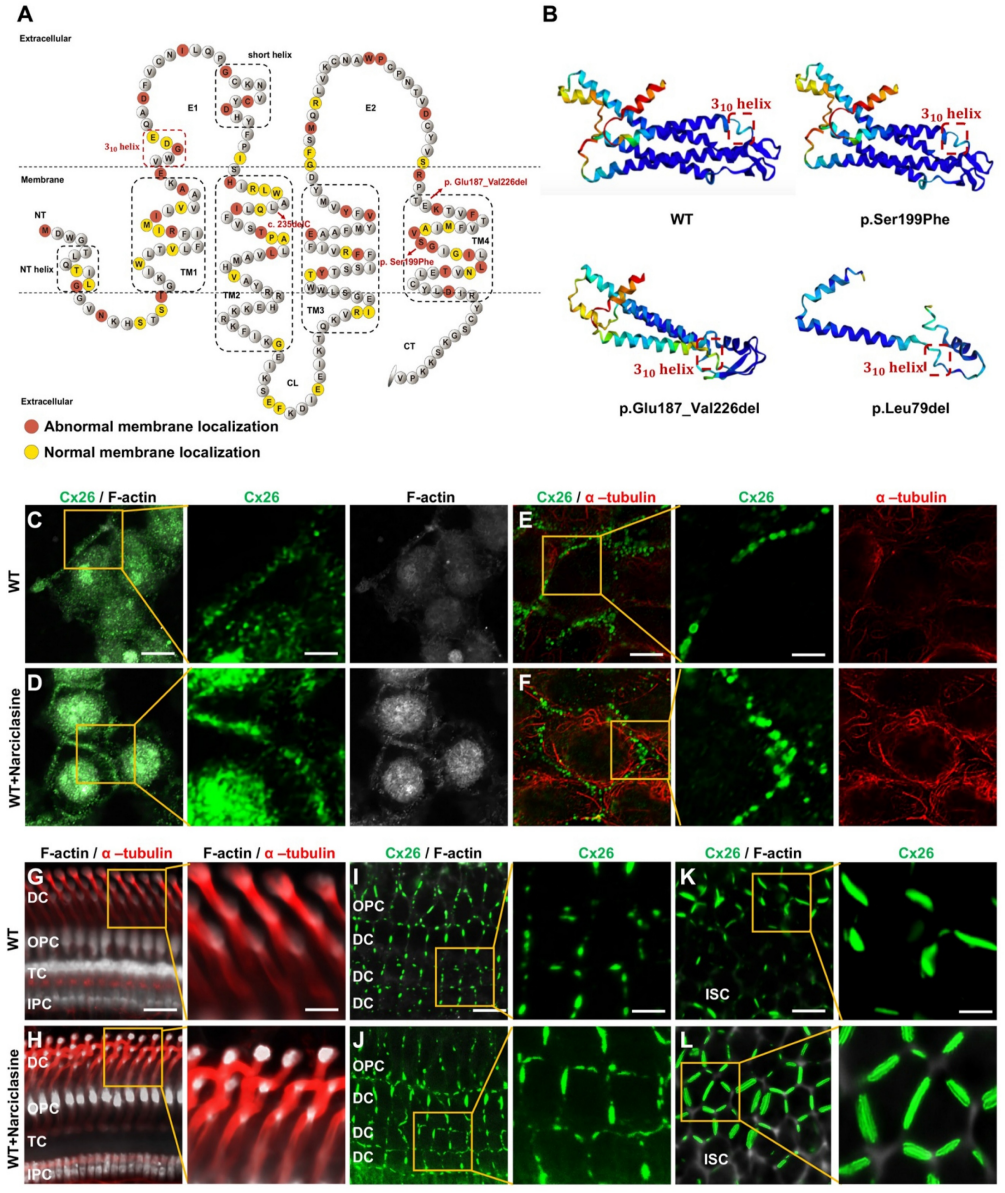
** Narciclasine promotes microtubules and actin microfilaments development and enhances the membrane localization of WT-Cx26 *in vitro* and *in vivo*. (A)** Schematic representation of the Cx26 structure and the Cx26 mutants retained in the cytoplasm. **(B)** Schematic representation of the three-dimensional structure of WT-Cx26 and Mut-Cx26s (p.Ser199Phe, p.Glu187_Val226del, and p.Leu79del). **(C-F)** Immunofluorescent staining of Cx26 (green), F-actin (white), and acetylated α-tubulin (red) in the control group (C, E) and in the Narciclasine-treated group (D, F) *in vitro*.** (G-L)** Immunofluorescent staining of Cx26 (green), F-actin (white), and acetylated α-tubulin (red) in the control group (G, I, K) and in the Narciclasine-treated group (H, J, L) *in vivo*. Scale bars: 10 μm (panels C-F), 3 μm (partial enlargement in panels C-F), 27 μm (panels G-H), 9 μm (partial enlargement in panels G-H), 20 μm (panels I-L), and 7 μm (partial enlargement in panels I-L).

**Figure 7:**
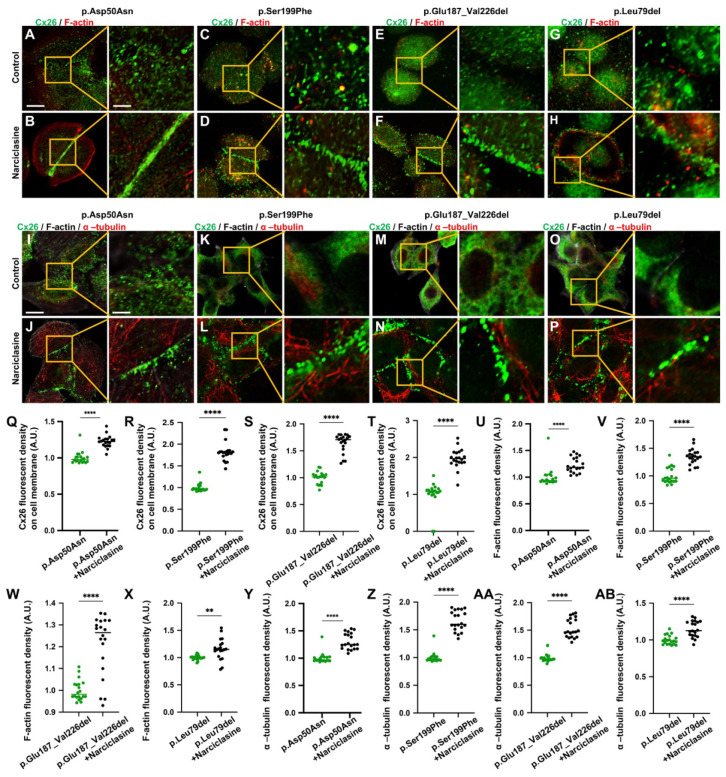
** Narciclasine promotes the development of cytoskeleton, and enhances the membrane localization of Mut-Cx26s *in vitro*. (A-H)** Immunofluorescent staining of Mut-Cx26s (green) and F-actin (red) in the Mut-Cx26 groups (A, C, E, G) and in the Narciclasine-treated groups (B, D, F, H) *in vitro*.** (I-P)** Immunofluorescent staining of Mut-Cx26s (green) and acetylated α-tubulin (red) in the Mut-Cx26 groups (I, K, M, O) and in the Narciclasine-treated groups (J, L, N, P) *in vitro*.** (Q-AB)** Quantification of the Cx26 fluorescent density on plasma membrane (Q-T), F-actin fluorescent density (U-X), and acetylated α-tubulin fluorescent density (Y-AB) from the Mut-Cx26 groups and the Narciclasine-treated groups *in vitro*. Scale bars: 10 μm (panels A-P), and 3 μm (partial enlargement in panels A-P). *P < 0.05, **P < 0.005, ***P < 0.001, ****P < 0.0001.

Previous research has indicated that specific high-frequency Cx26 mutants exhibited impaired transport to the plasma membrane, leading to their accumulation in intracellular organelles such as the Golgi apparatus and endoplasmic reticulum (ER). Nevertheless, the mechanisms underlying the abnormal membrane localization of these Cx26 mutants were still poorly understood. Analysis of data from the Deafness Genetic Database revealed that mutants with alterations in the TM4 region demonstrated the highest incidence of abnormal membrane localization, yielding a prevalence of 29.0% (Table 1). Consequently, three Mut-Cx26s associated with the TM4 region, namely p.Ser199Phe, p.Glu187_Val226del, and p.Leu79del (c.235delC), were hereby constructed, characterized by progressively reduced peptide lengths (Figure 6). Endeavors were made to determine the presence of abnormal membrane localization and relevant mechanisms of these three Mut-Cx26s (Figure 7A-AB) *in vitro*. As depicted in Figure 7C-H and Figure 7K-P, the Cx26 mutants all demonstrated diffuse retention within the cytoplasm and failed to form GJPs on the plasma membrane.

The development of cytoskeleton was further evaluated using immunofluorescence *in vitro*. FITC-phalloidin staining showed significant thinning of F-actin in the Mut-Cx26 groups (Figure 7V-X), further suggesting defective development of actin microfilaments arising from the mutants. Additionally, the results revealed reduced acetylated α-tubulin in these three Mut-Cx26s (Figure 7Z-AB), indicating that the mutants resulted in less microtubule stability. Concurrently, the above results confirmed significantly abnormal cytoskeleton development in the Mut-Cx26s *in vitro*, demonstrating abnormal cytoskeleton development as a possible reason for Mut-Cx26s exhibiting abnormal membrane localization and failing to form large GJPs on the plasma membrane.

**Figure 8:**
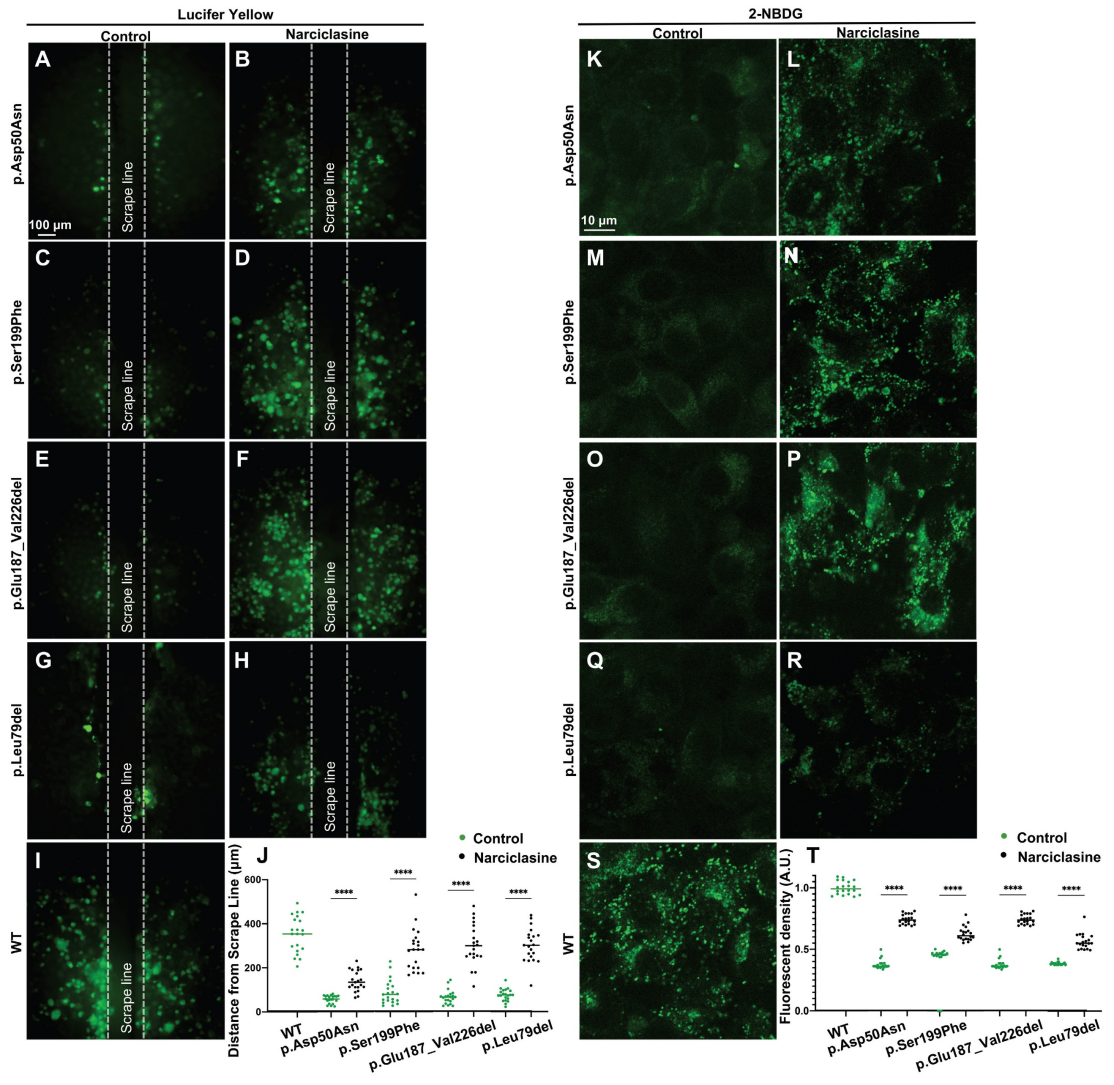
** Narciclasine increases permeability of cells to ions and glucose *in vitro*. (A-I)** Lucifer yellow diffusion images in scrape-loading dye transfer of WT-Cx26 (I) and Mut-Cx26s p.Asp50Asn (A-B), p.Ser199Phe (C-D), p.Glu187_Val226del (E-F), and p.Leu79del (G-H). **(J)** Quantification of the diffusion distance from the Mut-Cx26s and the Mut-Cx26s treated with Narciclasine. **(K-S)** 2-NBDG uptake in WT-Cx26 (S) and Mut-Cx26s p.Asp50Asn (K-L), p.Ser199Phe (M-N), p.Glu187_Val226del (O-P), and p.Leu79del (Q-R). **(T)** Quantification of the fluorescence intensity of 2-NBDG uptake from the Mut-Cx26s and the Mut-Cx26s treated with Narciclasine. *P < 0.05, **P < 0.005, ***P < 0.001, ****P < 0.0001.

Lucifer yellow (Figure 8A-J), a negatively charged dyes, can specifically diffuse through the gap junctions, facilitating its diffusion distance to reflect the ability of GJs-Cx26-mediated intercellular communication 48. 2-(N-(7-nitrobenz-2-oxa-1,3-diazol-4-yl)amino)-2-deoxyglucose (2-NBDG) (Figure 8K-T) represents a fluorescent analog of glucose that can be used to assess intercellular glucose transfer capacity 49, 50. Herein, Lucifer yellow and 2-NBDG were used to evaluate gap junction functions in the WT-Cx26 and in the Mut-Cx26s (p.Ser199Phe, p.Glu187_Val226del, and p.Leu79del) *in vitro*.

The results revealed a significant decrease in Lucifer yellow diffusion distance in the Mut-Cx26 groups (Figure 8C-H) compared with the control group (Figure 8I) *in vitro*. Meanwhile, the diffusion distance was reduced by 75.0%, 82.0%, and 79.0%, respectively (Figure 8J). As shown in Figure 8S, 2-NBDG fluorescence was primarily localized at the cell periphery in the WT-Cx26 group. The 2-NBDG fluorescence intensity was significantly reduced in the Mut-Cx26 groups (Figure 8M-R) *in vitro*, with the fluorescence intensity reduced by 60.7%, 63.0% and 60.0%, respectively (Figure 8T). These results indicated severely impaired functions of ion channels and nutrient transport in the Mut-Cx26s *in vitro*.

Collectively, these results indicated that the three Mut-Cx26s associated with the TM4 region possessed abnormal membrane localization and could not form large GJPs on the plasma membrane, thereby performing poor channel functions *in vitro*. The impaired membrane localization might be attributed to the abnormal cytoskeleton development.

### Narciclasine enhancing membrane localization of Cx26 *in vitro* and *in vivo*

Narciclasine stands out as a specific agonist for RhoA, a small G protein. Previous studies have shown that activation of RhoA promoted the development of the cytoskeleton, thereby facilitating the re-transport of some mutated membrane proteins to the plasma membrane. The three-dimensional structures of Cx26 mutants were previously predicted, and it was found that the three-dimensional structure of some Cx26 mutants did not change significantly (Figure 6B), with only the subcellular localization changed. This suggested that the sequence determining the subcellular localization of Cx26 and the sequence determining its structure and function were located in different regions, and that the structure and function of the mutants with subcellular localization changes might remain normal. As a result, it was hereby assumed that Narciclasine could possibly promote cytoskeleton development, facilitate the re-establishment of normal plasma membrane localization in Cx26 mutants retained in the cytoplasm, and restore the permeability of cells to ions and glucose.

Initially, validation was conducted in WT-Cx26 *in vitro* and *in vivo*. As shown in Figure 6C-F and Figure S1A-C, Narciclasine treatment resulted in a marked enhancement of acetylated α-tubulin and F-actin staining *in vitro*, whereas the Cx26 fluorescent density on the plasma membrane was significantly increased. Consistent findings were also obtained *in vivo*. In the Narciclasine-treated group, the staining of acetylated α-tubulin and F-actin was significantly enhanced in the supporting cells (Figure 6G-H, Figure S1D-E). Concurrently, the GJP lengths on the plasma membranes of DCs (Figure 6I-J, Figure S1F) and ISCs (Figure 6K-L, Figure S1G) were significantly increased.

Narciclasine was then adopted to treat cells transfected with the Mut-Cx26s (p.Ser199Phe, p.Glu187_Val226del, and p.Leu79del) for 3 h, and immunofluorescence staining was performed. As shown in the Figure 7A-AB, upon treatment with Narciclasine, the staining of F-actin (Figure 7C-H, Figure 7V-X) and acetylated α-tubulin (Figure 7K-P, Figure 7Z-AB) increased significantly. Compared with the control group, the fluorescence intensity on the plasma membrane of Mut-Cx26s increased 82.3%, 33.0%, and 22.8%, respectively (Figure 7C-H, Figure 7K-P, Figure 7R-T). This indicated that Narciclasine promoted the development of cytoskeleton and increased the membrane localization of Mut-Cx26s *in vitro*.

Lucifer yellow and 2-NBDG were also used to evaluate the ability of intercellular communication and glucose transfer following Narciclasine treatment *in vitro*. The Lucifer yellow diffusion distance in cells transfected with Mut-Cx26s p.Ser199Phe (Figure 8C-D), p.Glu187_Val226del (Figure 8E-F), and p.Leu79del (Figure 8G-H) was increased by 68.7%, 78.7%, and 75.0% upon treatment with Narciclasine compared with the non-treated group. Consistently, the 2-NBDG fluorescence intensity was also increased by 37.4%, 49.8%, and 31.7%, respectively, as shown in Figure 8M-R. These results indicated that Narciclasine enhanced the membrane localization of Mut-Cx26s, while increasing the permeability of cells to ions and glucose to a certain extent *in vitro*.

In summary, Narciclasine could promote the development of cytoskeleton, enhance the plasma membrane localization of the WT-Cx26 and the Mut-Cx26s retained in the cytoplasm, restore the permeability of cells to ions and glucose *in vitro*.

### Narciclasine rescuing hearing loss and hair cell degeneration in the *Gjb2^p.Asp50Asn/-^* heterozygous mice

**Figure 9:**
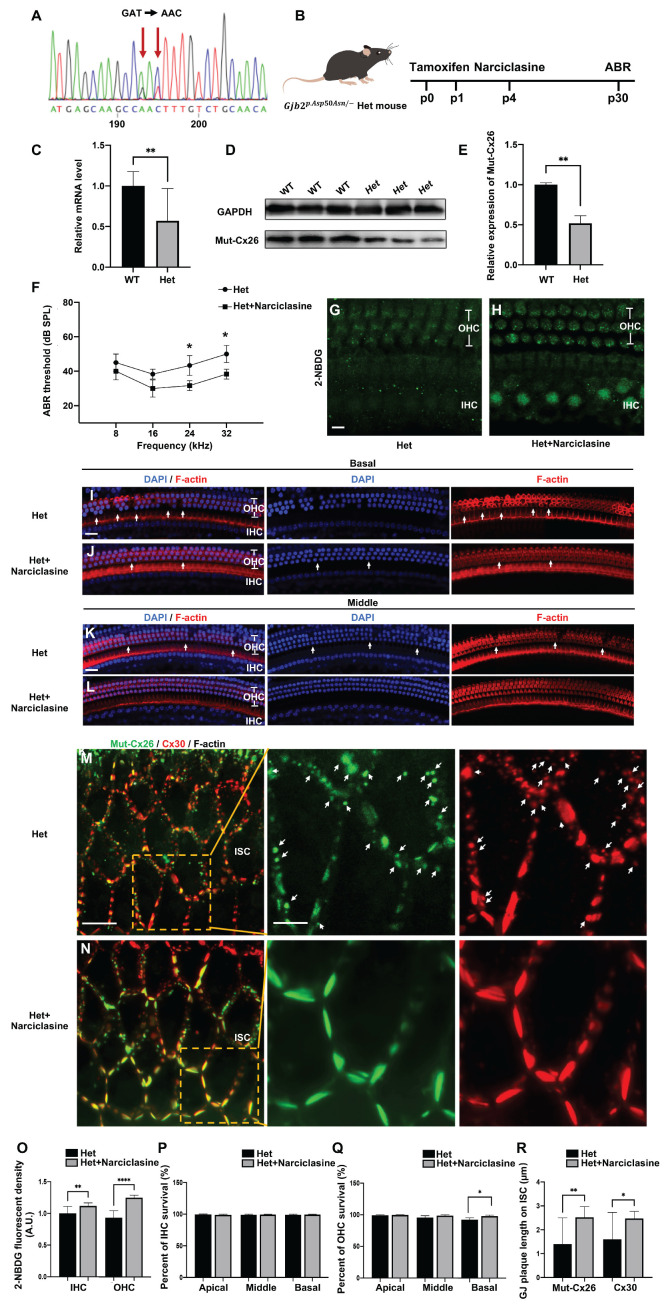
** Narciclasine rescues hearing loss and hair cell degeneration in the *Gjb2^p.Asp50Asn/-^* heterozygous mice. (A)** Sanger sequencing result of murine model. It confirms that the Het mouse harbors the p.Asp50Asn point mutation **(B)** Schematic diagram of the mouse operation process. **(C)** Changes in mRNA expression levels of *Gjb2* in the WT mice and *Gjb2* mutant in the Het mice. **(D-E)** Western blot and histogram showing the Cx26 protein levels in the WT mice and the Cx26 mutant protein levels in the Het mice. **(F)** Changes in ABR thresholds at different frequencies in the Het mice and in the Het mice treated with Narciclasine at P30 (*N*=5). **(G-H)** 2-NBDG uptake in the Het mice (G) and in the Het mice treated with Narciclasine (H) at P30. **(I-L)** Representative images of IHCs and OHCs of different turns in the Het mice (I, K) and in the Het mice treated with Narciclasine (J, L) at P30. **(M-N)** Immunofluorescent staining of Mut-Cx26 (green) and Cx30 (red) in the Het mice (M) and in the Het mice treated with Narciclasine (N) at P30. White arrows show Mut-Cx26 or Cx30 retained in the cytoplasm. **(O)** Quantification of the 2-NBDG relative fluorescence intensity from the Het mice and the Het mice treated with Narciclasine at P30.** (P-Q)** Percent of IHCs (P) and OHCs (Q) survival in the Het mice and in the Het mice treated with Narciclasine at P30. **(R)** Quantification of the length of GJPs on the plasma membrane of ISCs from the Het mice and the Het mice treated with Narciclasine at P30. Scale bars: 10 μm (panels G-H), 20 μm (panels I-L), 27 μm (panels M-N), and 9 μm (partial enlargement in panels M-N). *P < 0.05, **P < 0.005, ***P < 0.001, ****P < 0.0001.

Subsequently, experiments were carried out to assess the ability of Narciclasine to enhance the membrane localization of Mut-Cx26s and evaluate its potential to rescue hearing loss *in vivo* (Figure 9A-R). Given that mutations in the *Gjb2* gene were homozygous lethal in murine embryos 51, replicating the clinically observed pathological phenotype and exploring therapeutic interventions through the development of mutant murine models presents significant challenges. Previously, a *Gjb2^p.Asp50Asn/-^* heterozygous murine model (referred to as Het mouse) was successfully generated by the present research group utilizing CRISPR-Cas9 technology in conjunction with a conditional knockout mouse (unpublished data). Sanger sequencing confirmed that the Het mouse harbored the p.Asp50Asn point mutation (Figure 9A). Following tamoxifen administration (Figure 9B), the expression levels of Cx26 mutant in the Het mouse were found to be reduced by approximately 50% at both mRNA (Figure 9C) and protein levels (Figure 9D-E), indicating a complete knockout of wild-type Cx26, retaining only the Cx26 mutant.

In the Het mice treated with Narciclasine, the hearing thresholds exhibited a reduction of 11.7 ± 7.6 dB at 24 kHz and 13.3 ± 7.6 dB at 32 kHz, respectively (*N*=5, P<0.05, Figure 9F), compared with the untreated Het mice at P30. The glucose uptake capability of IHCs and OHCs was assessed using 2-NBDG, and a marked improvement was observed following Narciclasine treatment (Figure 9G-H, Figure 9O). Additionally, histological analysis indicated that Narciclasine mitigated the loss of OHCs in the basal turn of the Het mice (Figure 9I-L, Figure 9P-Q). Moreover, immunofluorescence staining was used to evaluate alterations in the membrane localization of the mutant (Figure 9M-N). In the Het mice, a portion of the Mut-Cx26 was retained in the cytoplasm (indicated by white arrows), involving a significant reduction and fragmentation of Mut-Cx26 on the plasma membrane. This severely hindered the formation of larger GJPs on the plasma membrane. Narciclasine treatment resulted in a significant increase in the presence of Mut-Cx26 on the plasma membrane, along with an increased length of the formed GJPs (Figure 9R).

These findings suggested that Narciclasine enhanced the membrane localization of Cx26 mutants, improved the glucose uptake capacity of hair cells, and effectively rescued hearing loss and hair cell degeneration *in vivo*, consistent with the outcomes observed in cellular experiments (Figure 7A-B, Figure 7I-J, Figure 8A-B, Figure 8K-L).

### Narciclasine rescuing hearing loss and hair cell degeneration in the Cx26 deletion mutation (Del-Cx26) mice

**Figure 10:**
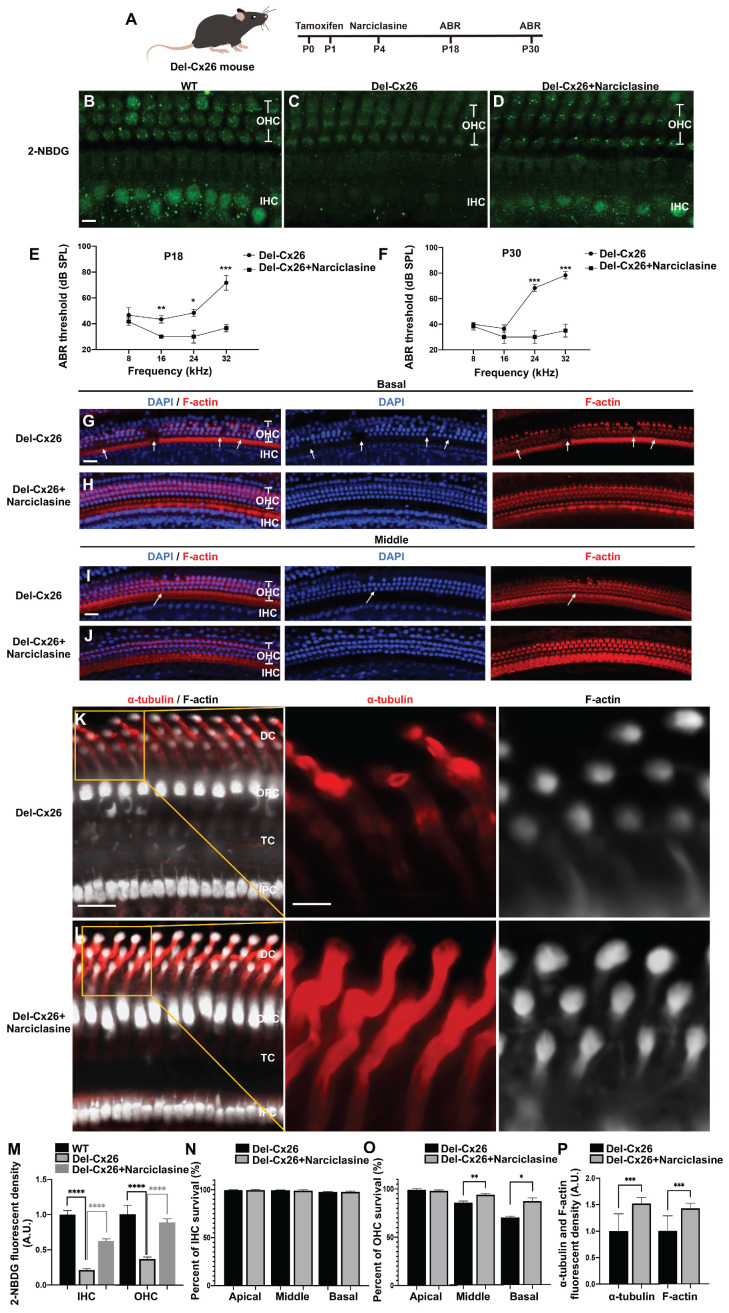
** Narciclasine rescues hearing loss and hair cell degeneration in the Del-Cx26 mice. (A)** Schematic diagram of mouse operation process.** (B-D)** 2-NBDG uptake in the WT mice (B), in the Del-Cx26 mice (C) and in the Del-Cx26 mice treated with Narciclasine (D) at P30. **(E-F)** Changes in ABR thresholds at different frequencies in the Del-Cx26 mice and in the Del-Cx26 mice treated with Narciclasine at P18 (E) and P30 (F) (*N*=10). **(G-J)** Representative images of IHCs and OHCs of different turns in the Del-Cx26 mice (G, I) and in the Del-Cx26 mice treated with Narciclasine (H, J) at P30. **(K-L)** Immunofluorescent staining of acetylated α-tubulin (red) and F-actin (white) in the Del-Cx26 mice (K) and in the Del-Cx26 mice treated with Narciclasine (L) at P30. **(M)** Quantification of the 2-NBDG relative fluorescence intensity from the Del-Cx26 mice and the Del-Cx26 mice treated with Narciclasine at P30. **(N-O)** Percent of IHCs (N) and OHCs (O) survival in the Del-Cx26 mice and in the Del-Cx26 mice treated with Narciclasine at P30. **(P)** Quantification of the acetylated α-tubulin and F-actin relative fluorescence intensity from the Del-Cx26 mice and the Del-Cx26 mice treated with Narciclasine at P30. Scale bars: 10 μm (panels B-D), 20 μm (panels G-J), 27 μm (panels K-L), and 9 μm (partial enlargement in panels K-L). *P < 0.05, **P < 0.005, ***P < 0.001, ****P < 0.0001.

As observed, a significant proportion of Cx26 mutants failed to reach the plasma membrane and served as models for inner ear gap junction deficiencies. Herein, a Del-Cx26 murine model mimicking truncated mutations was used to determine the rescue effect of Narciclasine on damaged hair cells and hearing loss (Figure 10A-P). Specifically, Narciclasine was injected into the unilateral ear through the round window injection technique, and the hearing was tested at P18 and P30 (Figure 10A). The ability of hair cells to uptake glucose was evaluated using 2-NBDG. As shown in Figure 10B-D and Figure 10M, the ability of IHCs and OHCs to take up glucose in Del-Cx26 mouse was significantly reduced and obviously improved, respectively, following Narciclasine injection.

Contrasting with the Del-Cx26 mice without any treatment, hearing thresholds of Del-Cx26 mice treated with Narciclasine showed mild decreased at 16, 24, and 32 kHz at P18, involving reductions of 13.3 ± 2.9 dB, 18.3 ± 7.6 dB, and 35.0 ± 8.7 dB, respectively (*N*=10, P<0.05, Figure 10E). Concurrently, significant hearing threshold reductions were observed at P30 at high frequencies, exhibiting improvements of 38.3 ± 7.6 dB and 43.3 ± 7.6 dB at 24 and 32 kHz, respectively *(N*=10, P<0.05, Figure 10F). Counting of OHCs along the entire cochlear spiral showed that Narciclasine reduced the loss of OHCs in the Del-Cx26 mice (Figure 10G-J, Figure 10O). In comparison, no significant loss of IHCs was observed either prior to or following the use of Narciclasine (Figure 10G-J, Figure 10N). Immunofluorescence staining was used to explore the mechanism of Narciclasine in rescuing hearing loss and hair cell degeneration. Upon Narciclasine treatment, the distribution of acetylated α-tubulin was more extensive, and the staining intensity was increased (Figure 10K-L, Figure 10P). Additionally, ortical actin was obviously thickened (Figure 10K-L, Figure 10P).

A reduction in the presence of Cx30 was observed on the plasma membrane of DCs in Del-Cx26 mice. This decrease might be attributed to the transdominant negative effect of the Cx26 mutation on Cx30 52. And the impaired translocation of Cx26 to the plasma membrane in turn disrupted the normal membrane localization of Cx30. Notably, treatment with Narciclasine resulted in a significant increase in Cx30 levels on the plasma membrane of DCs (Figure S2). Cx30 was highly expressed in the inner ear, where it served a critical channel function. The protein sequence identity between Cx30 and Cx26 was approximately 77% 53, and they were extensively co-expressed in the cochlea 54. Consequently, the enhanced plasma membrane localization of Cx30 following Narciclasine treatment might partially compensate for the functional deficits associated with Cx26. This could also provide insights into the mechanism underlying the therapeutic effect of Narciclasine on hearing loss in Del-Cx26 mice.

Intraperitoneal injection, a systemic administration method, was also used to assess the protective effects of Narciclasine on auditory function. In the Del-Cx26 mice treated with Narciclasine, the hearing thresholds exhibited a reduction of 16.7 ± 2.9 dB at 32 kHz (*N*=5, P<0.05, Figure S3A) compared with the untreated Del-Cx26 mice at P18. Meanwhile, the hearing thresholds were reduced by 15.0 ± 8.7 dB and 13.3 ± 7.6 dB at 24 kHz and 32 kHz, respectively (*N*=5, P<0.05, Figure S3B) at P30. Furthermore, hair cell count analyses indicated a decrease in the loss of OHCs in the middle and basal turns of the cochlea (Figure S3C-H). These findings suggested that intraperitoneal Narciclasine injection offered partial protection against hearing loss and hair cell degeneration in Del-Cx26 mice. However, its efficacy appeared to be inferior to that achieved through round window injection. This discrepancy might be attributed to the presence of the blood-inner ear barrier, which likely reduced drug concentration within the inner ear.

These results indicated significant protection provided by Narciclasine treatment against Cx26-deficiency-induced hearing loss and hair cell degeneration, which was likely achieved by promoting the development of the cytoskeleton and enhancing the membrane localization of Cx30.

## Discussion

The underlying mechanisms of the membrane localization of Cx26 remain a subject of controversy 55. Certain investigations have indicated that the membrane localization of Cx26 relies on the Golgi apparatus but is not dependent on microtubules 56. Conversely, other research has proposed that Cx26 may circumvent the Golgi apparatus and be transported to the plasma membrane through a microtubule-mediated vesicular transport mechanism 57, 58. Additionally, there is an ongoing debate regarding the potential dependence of Cx26 membrane localization on actin microfilaments. Given that Cx26 is devoid of a PDZ-binding motif and fails to exhibit co-localization with F-actin, some studies have put forward the hypothesis that its membrane localization is not reliant on actin microfilaments 58-60. However, there is evidence indicating that when actin microfilaments are disrupted, there is a 40% decrease in dye transfer through Cx26 60. Additionally, upon the knockdown of Cx26, a 54.85% reduction in F-actin can be observed 61. These findings have prompted some researchers to propose that the Cx26 membrane localization may actually be contingent upon actin microfilaments.

The dependence of the Cx26 membrane localization on cellular structures, including microtubules, actin microfilaments, and the Golgi apparatus, remains poorly understood. This uncertainty presents challenges in the potential for rescuing hearing loss through the modulation of the membrane transport pathway. In the present study, a comprehensive investigation of the membrane localization of Cx26 was conducted across three levels: cellular, tissue, and *in vivo*. The findings demonstrate the membrane localization of Cx26 as a multifaceted biological process that relies on the integrity and functionality of intracellular transport network consisting of microtubules, actin microfilaments, and the Golgi apparatus. This reliance may be attributed to the structural and functional interactions among these components 62, which collectively establish a network for membrane protein transport. For instance, the Golgi apparatus matters considerable in forming non-centrosomal microtubules 63, which are vital for the directional transport of vesicles to the plasma membrane. Additionally, microtubules are crucial for the distribution and localization of organelles and their depolymerization can lead to reversible fragmentation and dispersion of Golgi apparatus 64. Furthermore, microtubule depolymerization can trigger the reorganization of actin microfilaments, thereby extending actin microfilaments towards bicellular junctions 65. These interactions may account for the variability observed in laboratory results.

SPTBN1, responsible for encoding βII Spectrin, is crucial for the targeting of membrane proteins. Previous study has demonstrated that SPTBN1 can immunoprecipitate with various membrane proteins 66, 67, including Na^+^/K^+^ ATPase, Na^+^/Ca^2+^ exchanger, and αII Spectrin, as well as cytoskeleton elements such as actin, in both mouse and human cardiomyocytes. Furthermore, the knockdown of SPTBN1 reduces RyR2 and αII Spectrin on mouse cardiomyocytes membranes, subsequently leading to severe arrhythmias 66. This suggests that SPTBN1 may regulate the transport of membrane proteins by influencing the cytoskeleton framework and the localization of plasma membrane protein complexes. In the current investigation, findings from IP-MS, immunofluorescence, and Co-IP consistently indicate the interaction between SPTBN1 and Cx26. Notably, when SPTBN1 is knocked down, the membrane localization of Cx26 is severely disrupted. This observation highlights the crucial role of SPTBN1 in the process of Cx26 localizing to the membrane. Additionally, this study reveals a decrease in the co-localization of Mut-Cx26s linked to the TM4 region with SPTBN1. This indicates that the TM4 region may be the site where Cx26 and SPTBN1 interact with each other. The diminished ability of these mutants to interact with SPTBN1 may account for their abnormal membrane localization. Such a hypothesis warrants further investigation.

Previous research has established that the adherens junctions founded on N-cadherin play a significant role in the membrane localization of Cx26. Defourny *et al.* 44, 58, 68, 69, upon extensive investigations, have put forth a transport modeling hypothesis of Cx26. Specifically, they suggest that tricellular adherens junctions serve as a platform for the delivery of Cx26. N-cadherin facilitates the anchoring of microtubules to lipid rafts, which are believed to be located at tricellular adherens junctions. Consequently, Cx26 hemichannels are transported along these microtubules to lipid raft-rich regions at the cell surface. Considering the low affinity of Cx26 for cholesterol, the Cx26 hemichannels that are targeted to the lipid rafts undergo rapid lateral diffusion. As a result of this diffusion, these hemichannels accumulate at the periphery of preexisting GJPs.

N-cadherin represents an essential component of cellular processes. Its primary functions involve regulating microtubule dynamics and stabilizing microtubules, which are essential for Cx26 membrane localization. This mechanism facilitates the fusion of Cx26 transport vesicles with the plasma membrane at designated locations. Following the application of GC-4 68, an antibody that neutralizes N-cadherin, it has been observed that microtubules fail to anchor to the plasma membrane. This decreases the length of Cx26 GJPs and accumulates Cx26 within the cytoplasm. In summary, these observations underscore the significant role of N-cadherin-based adherens junctions in the proper membrane localization of Cx26. The augmentation of N-cadherin-based adherens junctions has the potential to drive the re-localization of both WT-Cx26 and Cx26 mutants to the plasma membrane. This redistribution event is instrumental in enhancing the functionality of their channels. Such findings present a promising avenue for future research with substantial potential for practical applications, necessitating further investigation.

Notably, while Cx26 GJPs do not co-localize with ephrin-B2 in the ISCs 70, 71, ephrin-B2 is specifically expressed in cochlear support cells. Research indicates that cell surface ephrin/ephrin interactions facilitate gap junction communication (GJC) 72, 73. Moreover, ephrin has been shown to affect the transport and degradation of Cx30 70, 74 and Cx43 72. Furthermore, ephrin-B2 harbors a PDZ-binding motif, which empowers it to assemble multiprotein complexes with other molecules that bear the PDZ domain, thereby participating in the endolymphatic potassium cycle. Consequently, the possible role of Ephrin/Eph as a significant regulatory protein in the membrane localization of Cx26 remains to be further investigated.

Herein, the probability of pathogenicity and membrane transport disorders associated with point mutations at various sites were analyzed based on the Deafness Genetic Database. As illustrated in Table 1, mutants located in the 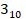
helix region exhibit the highest pathogenicity, indicating that this region has the closest correlation with the function of Cx26. Conversely, the region with the highest likelihood of membrane transport disorder is the TM4 region, which strongly indicates its crucial role in ensuring the correct membrane localization of Cx26. This implies that the sequences responsible for determining the subcellular localization of Cx26 and those that govern its structure and function may be located in distinct segments. Certain Cx26 mutants may only exhibit alterations in subcellular localization, while maintaining normal structural and functional characteristics. Consequently, the hypothesis was hereby proposed that enhancing the membrane localization of mutants retained in the cytoplasm may rescue hearing loss.

The present research further established a cell line expressing the p.Ser199Phe point mutation, situated in the TM4 region and prevalent in the human population 75. Structural prediction model shows that p.Ser199Phe shares a similar three-dimensional structure to wild-type Cx26 (Figure 6B). As indicated by cell experiments, p.Ser199Phe is retained in the cytoplasm, and can be re-transported to the plasma membrane and exert channel functions following Narciclasine treatment. Further, the research also confirms the effect of Narciclasine on the mutant with TM4 segment deletion. Subsequently, another cell line was built, which expresses the truncation mutation p.Leu79del, the most common variant in Asian species 3. The objective is to determine the effectiveness of Narciclasine for this mutant, which lacks a significant portion of the protein yet retains a 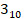
helix region analogous to that of the wild-type Cx26 (Figure 6B). Remarkably, Narciclasine enhances the membrane localization of p.Leu79del and partially restores the permeability of cells to ions and glucose. These findings demonstrate the possible therapeutic potential of Narciclasine for various point mutations or truncation mutations retained in the cytoplasm, or even missing mutations, thereby broadening its applicability.

The extensive reliance of the Cx26 membrane localization on microtubules, actin microfilaments, and the Golgi apparatus indicates that targeting a single transport pathway may be insufficient to attain the desired outcomes. Consequently, an agent capable of simultaneously enhancing all three transport pathways, namely microtubules, actin microfilaments, and the Golgi apparatus, should be necessarily identified to effectively promote the membrane localization of Mut-Cx26s retained in the cytoplasm, thereby restoring the permeability of cells to ions and glucose.

RhoA activation facilitates the development of microtubules, actin microfilaments, and the Golgi apparatus, and enhances intracellular membrane protein transport processes reliant on these structures. Previous study demonstrates that RhoA-mDia signaling promotes the assembly and stability of microtubules 76, which in turn allows for axon elongation. Additionally, Rab6 interacts with kinesin and dynein, thereby aiding in the transport of vesicles along microtubules 77, 78. Furthermore, RhoA activation has been shown to promote the polymerization of actin 79, the formation of actin stress fibers, and the translocation of the Tyrosine-protein kinase transforming protein v-Src from its site of synthesis (perinuclear) to the plasma membrane 80, 81. Previous studies indicate that Rab can interact with myosin V, thereby linking vesicles to actin microfilaments and facilitating their movement towards the cell surface 82, 83. Further studies also show that the deletion of the *RhoA* gene disrupts the structural integrity of Golgi apparatus, leading to its fragmentation and dispersion 84-87. Moreover, the recycling of CX3CR1 through the Golgi-derived vesicle pathway is also adversely affected 87.

The present findings corroborate prior studies. Narciclasine is hereby observed to induce microtubules and actin microfilaments development. As a consequence, the membrane localization of wild-type Cx26 is subsequently increased both *in vitro* and *in vivo*. This investigation is expanded to Mut-Cx26s. Narciclasine facilitates the cytoskeleton development and enhances the membrane localization of Mut-Cx26s retained in the cytoplasm *in vitro*. Furthermore, it promotes intercellular communication and glucose transfer.

**Figure 11:**
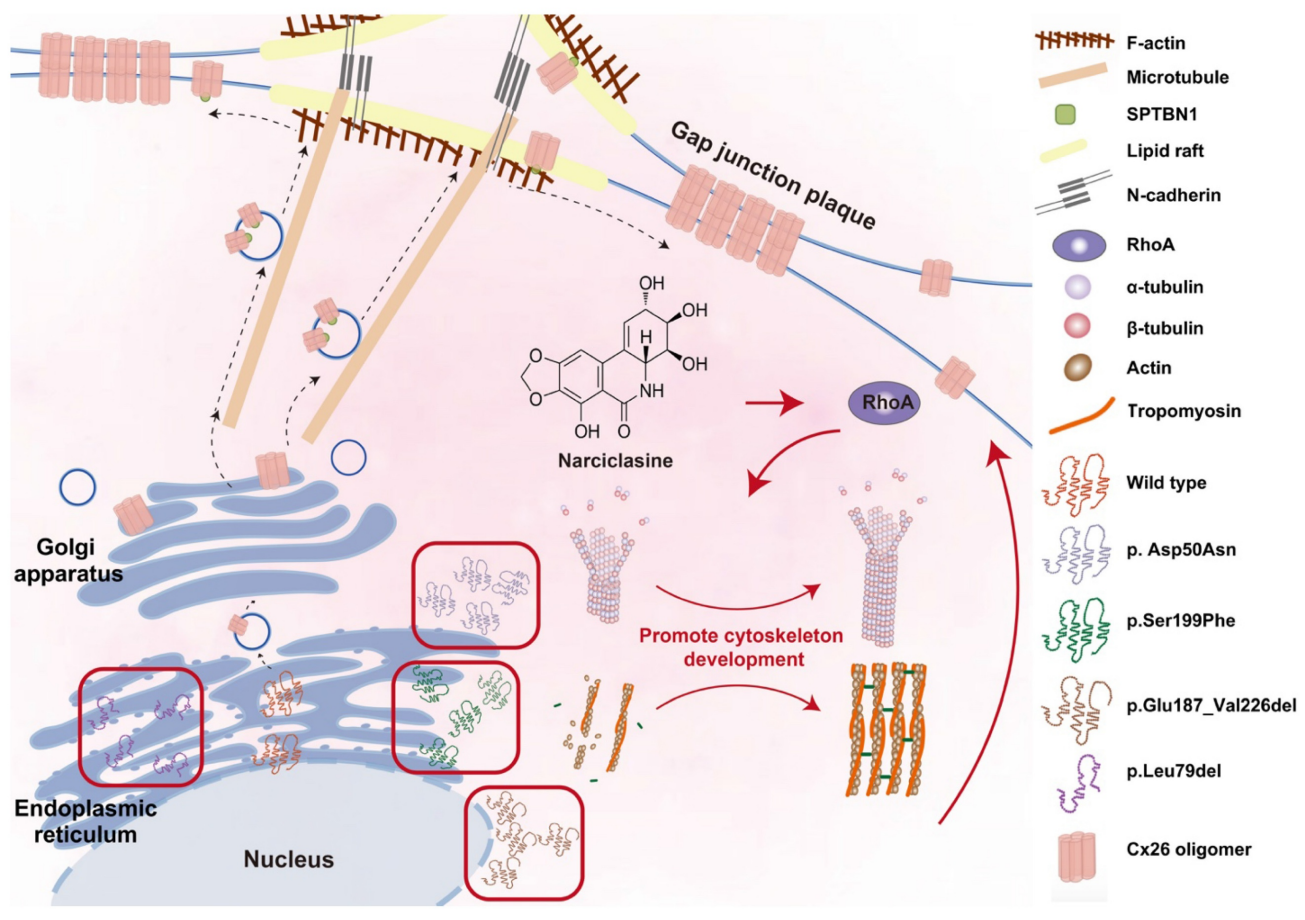
** Narciclasine enhances the membrane localization of Mut-Cx26 retained in the cytoplasm by facilitating the cytoskeleton development, thus restoring the permeability of cells to ions and glucose.** The membrane localization of WT-Cx26 relies on an intracellular protein transport network consisting of microtubules, actin microfilaments, and the Golgi apparatus. SPTBN1 is involved in the membrane localization of WT-Cx26. Tricellular adherens junctions serve as a platform for the delivery of Cx26 and N-cadherin facilitates the anchoring of microtubules to lipid rafts. Mut-Cx26 p.Glu187_Val226del retains in the perinuclear nucleus, while p.Asp50Asn, p.Ser199Phe and p.Leu79del retain in the ER. Narciclasine promotes the development of the cytoskeleton, thus enhancing the membrane localization of Mut-Cx26s retained in the cytoplasm. Black arrows represent WT-Cx26 membrane transport processes. Red arrows represent a diagram of the potential mechanism of Narciclasine enhancing the membrane localization of Mut-Cx26s to rescue hearing loss.

Herein, the findings were validated utilizing a murine model. The deficiency or point mutation of Cx26 has been shown to impact glucose transport within the placenta of mouse models, resulting in embryonic mortality. Consequently, the development of such animal models presents significant challenges 55. In the prior investigations conducted by the present research group, a heterozygous mouse model was successfully developed. As suggested by the observations, the Mut-Cx26 within this heterozygous mouse model is retained in the cytoplasm, while the presence of Mut-Cx26 in the plasma membrane is markedly reduced and fragmented. These findings are consistent with cellular data. They suggest that the heterozygous mouse model effectively replicates clinical instances of Cx26 point mutations. In these cases, membrane localization and channel functionality associated with hearing loss are impaired. In agreement with cellular experimental outcomes, a single round-window injection of Narciclasine leads to a substantial enhancement of Mut-Cx26 on the plasma membrane, thereby forming larger GJPs (Figure 9M-N). The present experimental findings confirm that Narciclasine enhances the membrane localization of Mut-Cx26 retained in the cytoplasm while increasing the permeability of cells to ions and glucose at both *in vitro* and *in vivo* levels. As a consequence, hearing loss and hair cell degeneration are rescued to a certain degree (Figure 11).

In murine models, deletions or point mutations in the *GJB2* gene are inherently lethal. Coupled with the constraints of current reproductive technologies, this presents a considerable obstacle to the acquisition of homozygous mutant progeny 31, 32, 88, 89. Concurrently, the establishment and breeding of a heterozygous murine model is equally intricate. At present, the therapeutic effects of Narciclasine can only be validated in two specific animal models, namely the Fgfr3-iCreER^T2^; Cx26^loxp/loxp^ murine model and the *Gjb2^p.Asp50Asn/-^* heterozygous murine model. This is a limitation faced by the present study. Overall, this research provides robust evidence confirming the effectiveness of Narciclasine in rescuing hearing loss and hair cell degeneration. These conditions are caused by either a point mutation in Cx26 or its reduced expression, both of which result in decreased Cx26 levels on the plasma membrane. This discovery significantly broadens the potential applications of Narciclasine.

Impaired ion exchange and glucose supply, resulting from the absence or dysfunction of gap junctions, may contribute to the development of hearing loss 16, 90-92. A single injection of Narciclasine into the round window of mice has been shown to rescue hearing loss and restore hair cell function. Herein, an innovative approach to hearing preservation was proposed. This approach entails promoting the recovery of membrane transport process in mutant models. Additionally, in involves repairing and reconstructing the gap junction network and restoring its functionality within the inner ear. This perspective offers a novel therapeutic strategy for addressing *GJB2*-associated hereditary sensorineural hearing loss.

## Methods

### Cell culture

HEK293T and Hela cells were cultured in Dulbecco's modified Eagle's medium (DMEM, HY2010, HY CEZMBIO) supplemented with 10% fetal bovine serum (FBS) (FBS500-H, HY CEZMBIO) and 1% antibiotics (HYG2222, HY CEZMBIO), and maintained at 37 °C in a humified incubator with 95% air and 5% CO2. When the cells grow to a cell confluence of about 80-90%, perform cell passaging.

### Transfections and generation of stable cell lines

WT-Cx26 and Cx26 mutants p.Asp50Asn, p.Ser199Phe, p.Glu187_Val226del, and p.Leu79del were prepared as described 93. The recombinant viral plasmid encoding lentiviral particles and its helper packaging agent vector plasmid were prepared, the recombinant plasmid and helper plasmid were extracted with high purity and endotoxin-free, and HEK-293T cells were co-transfected with GM 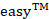
Lentiviral Packaging Kit (GMeasy-10, Genomeditech). Cells were inoculated into 10 cm dishes and transfected when cell fusion reached 80%. Fresh medium was changed 1-2 h before transfection. Take a sterile 1.5 ml EP tube, add 1 ml serum-free DMEM medium, 10 μg DNA, 10 μl GM easy^TM^ Lentiviral Mix and 60 μl HG Transgene^TM^ Reagent to form a reaction system. After mixing, let it stand at room temperature for 18-20 min, and then add it evenly to the petri dishes. After 18-20 h of transfection, the culture was replaced by fresh 15 ml serum-free DMEM medium. After 48 h, the supernatant was aspirated into a centrifuge tube and centrifuged at 500 g for 5 min at 4 °C. The supernatant was filtered through a 0.45 μm filter and transferred to a new centrifuge tube, and stored at -80 °C. For cell infection, cells were inoculated in 6-well plates, and the collected viral fluid was added to the cells when the cell fusion reached 60%. The medium was changed after 24 h of infection, and the expression of target proteins was detected by Western blotting or immunofluorescence at 96 h after the medium change.

### Transfection of siRNA silences gene expression

Cells were inoculated in six-well plates at a density of 10^5 cells per well, and transfection was performed when cell fusion reached 50-60%. Configure the transfection system in RNase Free EP tubes. Mix 125 μl DMEM medium and 5 μl lipofectamine 3000 (L3000001, ThermoFisher Scientific) by gentle blowing as System 1. Mix 125 μl DMEM medium and 5 μl SPTBN1 siRNA or negative control siRNA by gentle blowing as System 2. Stand for 5 min. Mix System 1 and System 2 well, let it stand for 15-20 min, and add it evenly to the cell wells containing 1 ml of DMEM medium. 6-8 h after transfection, the medium was changed to complete medium containing 1% FBS. After 96 h of the medium change, differences in protein expression levels were detected by Western blotting.

### Drug treatments

Cells were treated with the following drugs dissolved in culture media: 0.02 mg/ml nocodazole (HY-13520, MCE) for disruption of microtubules; 0.001 mg/ml cytochalasin B (HY-16928, MCE) for disruption of actin microfilaments; 0.005 mg/ml BFA (HY-16592, MCE) for disruption of Golgi apparatus. For cochlear explants, the drug concentration used was 2 mg/ml nocodazole, 0.015 mg/ml cytochalasin B, and 0.05 mg/ml BFA. For *in vivo* round window injections in mice, the drug concentration used was 0.4 mg/ml nocodazole, 0.4 mg/ml cytochalasin B, and 0.2 mg/ml BFA.

### Mouse model and genotyping

The Cx26^loxp/loxp^ mice were provided by Prof. Xi Lin of Emory University, Atlanta, GA, USA, and the Fgfr3-iCreER^T2^ mice were provided by Prof. Zhiyong Liu of the Chinese Academy of Sciences, Shanghai, China. As previously reported 38, 94, adult Cx26^loxp/loxp^ mice were crossed with Fgfr3-iCreER^T2^ mice to generate first generation Fgfr3-iCreER^T2^; Cx26^loxp/WT^ mice. The Fgfr3-iCreER^T2^; Cx26^loxp/WT^ offspring were backcrossed to generate Fgfr3-iCreER^T2^; Cx26^loxp/loxp^ mice. The Fgfr3-iCreER^T2^; Cx26^loxp/loxp^ mice were the experimental group and the Cx26^loxp/loxp^ littermates were the control group. Two consecutive subcutaneous injections of tamoxifen (TMX) (total dose 1.5 mg/10 g body weight, T5648, Sigma-Aldrich), once on P0 and again on P1, were administered to specifically activate Cre recombinase and target Cx26-null in early cochlear PCs and DCs. And the *Gjb2^p.Asp50Asn/-^* heterozygous mice were obtained by CRISPR-Cas9 and crossed with the Rosa26-CreER; Cx26^loxp/loxp^ mice (unpublished data). Mouse genotyping was carried out following polymerase chain reaction (PCR) amplification of genomic DNA from tail tissue as previously described 95. Mice were maintained on ad libitum chow and high pressure filtered water and were kept at a constant temperature (21 °C-25 °C), 40%-70% humidity, and 24 h day/night light cycle. All procedures were approved by and conducted in accordance with the guidelines of the Animal Care and Use Committee of Tongji Medical College, Huazhong University of Science and Technology. The primer sequences used are listed below.

*Cx26 (F)*: 5′-ACAGAAATGTGTTGGTGATGG-3′,

*Cx26 (R)*: 5′-CTTTCCAATGCTGGTGGAGTG-3′,

*Fgfr3iCreER (F)*: 5′-GAGGGACTACCTCCTGTACC-3′,

*Fgfr3iCreER (R)*: 5′-TGCCCAGAGTCATCCTTGGC-3′.

### Auditory brainstem response (ABR)

ABR were used to assess the hearing thresholds at P18 and P30. The measurements were conducted using the Tucker-Davis Technology (TDT) System, as preciously detailed 38. Briefly, the mice were anesthetized and placed on a thermostatic electric blanket maintained at 37 °C. The recording and reference electrodes were inserted subcutaneously into the skull or the tested ear, respectively. Frequencies of 8, 16, 24, and 32 kHz were evaluated. The SigGen32 software (Tucker-Davis Technologies) was used to amplify and record the ABR signals across the specified frequencies.

### RNA preparation and real-time quantitative polymerase chain reaction

Total RNA was extracted from cochlear tissues utilizing an RNAprep pure tissue kit (Tiangen Biotech Co. Ltd.). The concentration and purity of the RNA were assessed using a NanoDrop 1000 spectrophotometer (NanoDrop). Subsequently, RNA was reverse-transcribed employing a PrimeScript RT reagent kit with gDNA eraser (Takara Bio Inc.). Real-time PCR was conducted using SYBR Green PCR mix on a Roche Light-Cycler 480 instrument. The 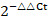
method was utilized to quantify relative mRNA expression. The primers used in this study are detailed below.

*Gjb2-WT (F)*: 5′-CTCGGGGGTGTCAACAAACA-3′, *Gjb2-WT (R)*: 5′-CACGAGGATCATGATGCGGA-3′, *Gjb2-Mut (F)*: 5′-TTGTCACCTATCAGCAGCCTAGAGG-3′, *Gjb2-Mut (R)*: 5′-TTTCATGTCTCCGGTAGGCCA-3′, *GAPDH (F)*: 5′-GAAGGTCGGTGTGAACGGAT-3′, *GAPDH (R)*: 5′-CTCGCTCCTGGAAGATGGTG-3′.

### Protein extraction and Western blot

Membrane proteins were extracted from cells or basement membranes using Membrane and Cytosol Protein Extraction Kit (P0033, Beyotime). Total proteins were extracted using RIPA lysis buffer (P0013B, Beyotime) or Cell lysis buffer for Western and IP (P0013, Beyotime) containing 1% PMSF (HYP112, HY CEZMBIO). Samples with equal amounts of protein were separated by 10% sodium dodecyl sulphate-polyacrylamide gel electrophoresis (SDS-PAGE) and then transfused onto polyvinylidene difluoride (PVDF) membranes. The PVDF membranes were blocked in blocking buffer prepared from tris-buffered saline (TBST containing 0.1% Tween-20) with 5% milk for 1 h at room temperature, followed by incubation with primary antibodies at 4 °C overnight. The primary antibodies used were: anti-Cx26 (1:1000 dilution, 710500, Invitrogen), anti-P-cadherin (1:1000 dilution,), anti-SPTBN1 (1:1000 dilution, 19722-1-AP, Proteintech), and anti-GAPDH (1:1000 dilution, ANT324, Antgene). The PVDF membranes were then incubated with horseradish peroxidase-conjugated goat anti-rabbit secondary antibody (1:5000 dilution, ANT019, Antgene) or rabbit anti-goat secondary antibody (1:5000 dilution, ANT, Antgene) for 1.5 h at room temperature. The bands were visualized using an extra sensitive electrochemiluminescence (ECL) reaction kit (MA0186, Meilunbio). Protein levels were measured using ImageJ software (National Institutes of Health) and normalized to P-cadherin (for membrane protein) or GAPDH (for total protein) levels in the corresponding lanes.

### Immunoprecipitation-mass spectrometry (IP-MS)

Cells that overexpressed Cx26 or wild-type mouse basement membranes were subjected to lysis using a Cell Lysis Buffer for Western and IP (P0013, Beyotime), supplemented with 1% PMSF (HYP112, HY CEZMBIO), and maintained at 4 °C for a duration of 30 min. Following lysis, the samples were centrifuged at 12,000 g for 10 min, after which the supernatant was incubated with Protein A/G Immunoprecipitation Magnetic Beads (B23201, Selleck) and anti-Cx26 antibody (138100, Invitrogen) at 4 °C overnight. The magnetic beads were subsequently washed three times with Wash Buffer. The mass spectrometry (MS) analysis was conducted by Seqhealth Technology Co., Ltd., Wuhan, China. MS raw data were subjected to analysis utilizing MaxQuant (V1.6.6) in conjunction with the Andromeda database search algorithm. Proteins that could not be differentiated based on unique peptides were consolidated by MaxQuant into a singular protein group. The search results were subsequently filtered to achieve a false discovery rate (FDR) of 1% at both the peptide and protein levels.

### Co-immunoprecipitation (Co-IP)

Using ProteinA/G Immunoprecipitation Magnetic Beads (B23201, Selleck) for Co-IP. A portion of the extracted protein was set aside as Input, and 2 μg anti-Cx26 (138100, Invitrogen) was added to the remaining protein extract, gently blown to mix, and then placed in a 4 °C turning shaker to incubate overnight. For magnetic bead pretreatment, 20 μl of magnetic beads were aspirated, washed with Wash Buffer for 2 min, and magnetically separated using a magnetic rack. The washing step was repeated three times. Antigen-antibody complexes were added to the pretreated magnetic beads and incubated overnight at 4 °C on a turning shaker to bind the magnetic beads to the antigen-antibody complexes. The binding product was magnetically separated, the supernatant was aspirated and stored in portions as Output. The antigen-antibody-magnetic bead complex was washed three times using Wash Buffer for 2 min each time and the supernatant was removed by magnetic separation. Add 50 μl of 1x SDS-PAGE Protein Sample Loading Buffer (P0287, Beyotime) to the wash product. Mix well and heat at 95 °C for 5 min to perform antigen elution. After heating, magnetic separation was performed and the supernatant was aspirated as the IP product. Detection of destination and target proteins in Input, Output, and IP samples was performed by Western blotting.

### Cell or cochlear tissue preparation and immunofluorescent labelling

Cells were fixed with 4% paraformaldehyde for 0.5 h at room temperature, then blocked in phosphate buffered saline (PBS) containing 1% bovine albumin (BSA) and 0.1% Triton X-100 solution for 15 min and in PBS containing 1% BSA for 45 min at room temperature. Cochlear dissection was carried out in euthanised mice. Briefly, the temporal bone was harvested and dissection was careful. To enable proper fluid exchange during immersion fixation in 4% paraformaldehyde for 1 h at room temperature, a small incision was made at the apical tips, followed by decalcification in ethylenediaminetetraacetic acid (EDTA) for 48 h until the cochlear bone wall was transparent. The cochleae were dissected under the microscope in three rotations (apical, middle and basal). The flattened cochlear preparations were blocked in PBS containing 1% BSA and 0.1% Triton X-100 solution for 1 h at room temperature. Cells or cochlea were incubated overnight at 4 °C with anti-Cx26 (1:300 dilution, 710500 or 138100, Invitrogen), anti-SPTBN1 (1:300 dilution, 19722-1-AP, Proteintech), anti-α-tubulin (K40 acetylated) (1:300 dilution, ab24610, Abcam) and anti-GM130 (1:300 dilution, 11380-1-AP, Proteintech). Samples were washed three times in PBS and then stained with Alexa Fluor 488 donkey anti-mouse IgG and Alexa Fluor 647 donkey anti-rabbit IgG (1:300 dilution, ANT033, ANT032) for 2 h. 4′,6-Diamidino-2-phenylindole (DAPI) (C1005, Beyotime) and phalloidin (0.05 mg/mL, P5282, Sigma-Aldrich) were used for nuclear and F-actin staining. A laser scanning confocal microscope (Nikon) was used for image acquisition. Confocal images are produced through the scanning of various layers of the sample along the Z-axis, followed by the superimposition of the collected layers.

The expression of interesting proteins was assessed by semi-quantification of immunocytochemical signals. Each image was taken under the same conditions. Quantitative analysis was performed using ImageJ software. All cochlear surface preparations were stained with phalloidin (red) or DAPI (blue) to identify OHCs or OHC cell nuclei in the confocal images. The intensity of the background was subtracted and then the average grey scale intensity was calculated for each cell. A total of 48-60 OHCs from four or five individual mice were counted for each region. Relative fluorescence was quantified by normalizing the ratio of the average fluorescence of target cells in the treatment group to that in the control group. The length of GJPs on the plasma membrane was measured by ImageJ software.

### Cochlear explant culture

The collagen gel was synthesized using the following procedure: rat tail collagen (Type 1, BD Biosciences) was carefully combined with 10× Eagle's Basal Medium (BME, B9638, Sigma) and 2% sodium carbonate in a volumetric ratio of 9:1:1 96-100. Subsequently, 10 μl of the collagen gel was deposited at the center of a 35 mm Petri dish and allowed to set for a duration of 30 to 60 min, after which it was overlaid with 1.0 ml of a culture medium. This medium was formulated to include 47.7 ml of 1 × BME (B-1-160, Sigma), 0.5 g of bovine serum albumin (BSA; A-4919, Sigma), 0.1 ml of penicillin G (P-3414, Sigma), 0.5 ml of Serum-Free Supplement (I-1884, Sigma), 0.5 ml of a 200 mM glutamine solution (G-6392, Sigma), and 1.2 ml of a 20% glucose solution (G-2020, Sigma).

C57BL/6 wild-type mice were euthanized at P5. Following a swift resection of the temporal bone, cochlear and vestibular explants were extracted and subsequently placed in ice-cold 1x Hank's balanced salt solution (BL561A, Biosharp). The cochlear basilar membrane, which connects the SGNs, and the macula of the utricle, which connects the superior vestibular ganglion neurons (VGNs), were meticulously dissected. The tectorial membrane and otolithic membrane were carefully removed, and the explants were positioned on a collagen gel substrate covered with culture medium. The cochlear and vestibular explants were incubated at 37 °C in a 5% CO2 atmosphere for a duration of 4 to 6 h to facilitate their adherence and growth on the collagen gel surface. Subsequently, an additional 1.0 ml of medium was introduced into the culture dish to support overnight growth of the explants under the same incubatory conditions. On the following day, the cochlear explants were subjected to treatment with nocodazole, Cytochalasin B, and BFA.

### Round window injection in neonatal mice

The P4 mice were cryogenically anesthetized under appropriate conditions. Anesthetized mice were placed in a cold ice box, and the area behind the left ear was disinfected with iodophor and deiodinated with 75% alcohol. Under a stereomicroscope, a 1 cm semilunar skin incision was made behind the left ear to bluntly separate the subcutaneous fat and muscle layers. After exposing the otocyst, a hole was made in the posterior upper quadrant of the auditory vesicle to expose the round window. A glass electrode was inserted into the round window using a three-dimensional manipulator and a 2 ul volume of drug was slowly injected using a hydraulic propeller. After the injection was completed and the pressure in the membrane cochlea was stabilized, the glass electrode was removed. The wound was closed layer by layer and sterilized with iodophor.

### Scrape-loading dye transfer *in vitro*

The evaluation of intercellular coupling mediated by gap junctions was conducted through the transfer of lucifer yellow dye utilizing a scrape-loading assay, as previously described with minor modifications 48. In summary, astrocytes were cultured in 12-well plates and maintained at 37 °C in a humidified atmosphere containing 5% CO2. Only those cells exhibiting 95-100% confluence were selected for the experiments detailed herein. The cells were incubated in a medium containing Narciclasine for a duration of 2 h. Following this incubation, the plates were subjected to three gentle washes with phosphate-buffered saline (PBS). A sterile 5 ml syringe tip was employed to create a cell-free area approximately 0.5 mm wide by scratching the astrocyte monolayers, thereby facilitating the entry of the dye into the cells. Subsequently, 1 ml of 0.4% lucifer yellow was introduced and incubated at 37 °C for an additional 30 min in the growth medium to permit the transfer of the loaded dye to adjacent cells. Excess dye was removed through two rinses with FBS-free DMEM. The cells coupled by gap junctions were visualized using a fluorescence microscope, and the diffusion distance of lucifer yellow was quantified using Image J software. Three random images were captured for each plate, and the means of these measurements were calculated.

### *In vitro* cells and *in vivo* mouse basement membrane hair cells glucose uptake assay

The capacity of cells to uptake glucose was assessed utilizing the fluorescent glucose analog 2-NBDG (ST2078, Beyotime) 101, 102. Cells were cultured in confocal dishes within DMEM medium devoid of glucose or other carbon sources. Following a 2 h treatment with Narciclasine, the cells were gently rinsed with DMEM medium and subsequently incubated with 0.2 mM 2-NBDG at 37 °C for a duration of 60 min. After incubation, the cells underwent three washes with DMEM medium. In the *in vivo* mouse model, the subjects were anesthetized via intraperitoneal administration of 1.25% tribromoethanol at a dosage of 0.2 ml per 10 g of body weight. A needle was subsequently introduced into the left ventricle, through which 0.2 ml of a 3.8 mM solution of 2-NBDG was administered. Following a five-minute interval, the apical turned-basement membranes were excised and prepared into spreads for further analysis. The fluorescence signal was then measured using laser confocal microscopy, with excitation at 465 nm and emission at 540 nm.

### Statistical information

All data are presented as mean ± SEM. GraphPad Prism 9.0.0 software (GraphPad Software Inc., San Diego, CA, USA) was used to generate graphs, and significance analyses were conducted by one-way ANOVA followed by Dunnett's test using SPSS 26.0 (SPSS, Chicago, USA).

## Supplementary Material

Supplementary figures.

## Figures and Tables

**Table 1:** Pathogenicity ratio and abnormal membrane localization ratio of Mut-Cx26s occurring in different segments of Cx26.

Domain		Pathogenicity ratio (recessive)	Membrane transport disorder ratio
NT		10% (6%)	9.1%
TM1		13% (12%)	14.3%
E1	3_10_ helix in E1	30% (12%)	16.7%
	E1 except 3_10_ helix	11% (3%)	18.2%
TM2		18% (16%)	10.8%
CL		1% (1%)	0%
TM3		6% (4%)	11.4%
E2		14% (11%)	18.5%
TM4		11% (10%)	29.0%
CT		0% (0%)	0%
AVERAGE		10% (8%)	16.4%

Abbreviations: NT, amino termini; TM1, transmembrane helix 1; E1, extracellular loop 1; TM2, transmembrane helix 2; CL, cytoplasmic loop; TM3, transmembrane helix 3; E2, extracellular loop 2; TM4, transmembrane helix 4; CT, carboxyl termini.
