# Supplementary material for: Promotion of Cx26 mutants located in TM4 region for membrane translocation successfully rescued hearing loss
Source: Theranostics. 2025 Apr 22;15(12):5801–25. doi: 10.7150/thno.112225 (PMC12068290; doi:10.7150/thno.112225)
Supplement: Supplementary file 1 — Supplementary figures. [file thnov15p5801s1.pdf]

Promotion of Cx26 mutants located in TM4 region for membrane translocation successfully rescued hearing loss

Yan-Jun Zong,[1†] Xiao-Zhou Liu,[1†] Xin-Yu Shi,[1] Zheng-Dong Zhao,[1] and Yu Sun[1,2,3,*]

1 Department of Otorhinolaryngology, Union Hospital, Tongji Medical College, Huazhong University of Science and Technology, Wuhan 430022, China.

2 Institute of Otorhinolaryngology, Union Hospital, Tongji Medical College, Huazhong University of Science and Technology, Wuhan 430022, China.

3 Hubei Province Clinic Research Center for Deafness and Vertigo, Wuhan 430022, China.

* Correspondence: sunyu@hust.edu.cn

† These authors contributed equally to this work

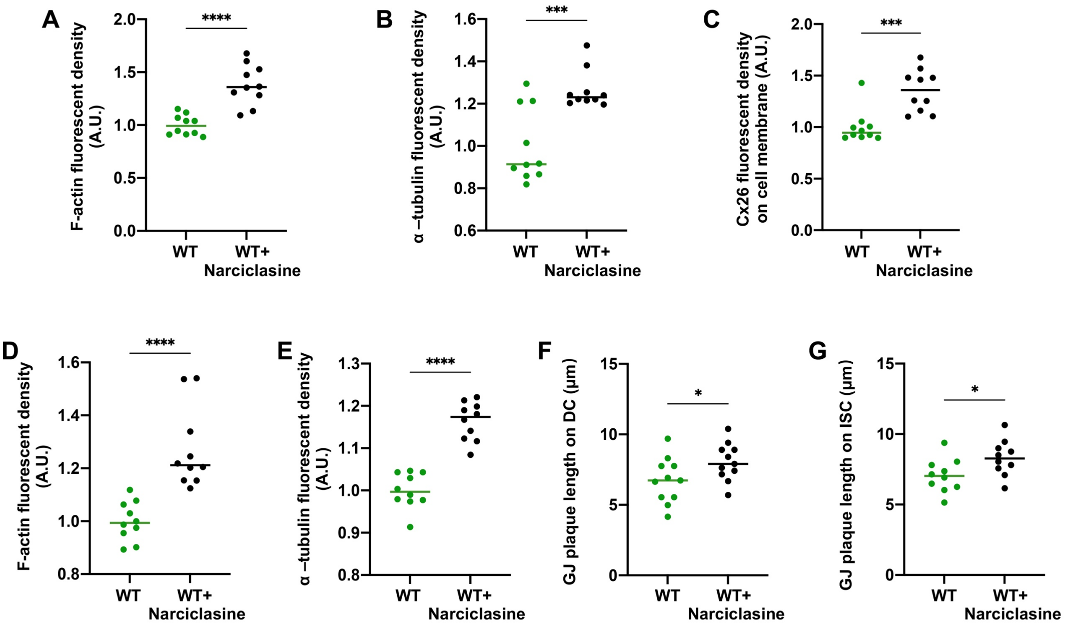

Figure S1 Narciclasine promotes the development of cytoskeleton and increases the membrane localization of WT-Cx26 in vitro and in vivo. (A-C) Quantification of the F-actin fluorescence intensity (A), acetylated α-tubulin fluorescence intensity (B), and Cx26 fluorescence intensity on plasma membrane (C) from the control group and Narciclasine-treated group in vitro. **(D-G)** Quantification of the F-actin fluorescence intensity (D), acetylated α-tubulin fluorescence intensity (E), and the lengths of GJPs on DCs (F) and ISCs (G) from the control group and the Narciclasine-treated group in vivo. $*P < 0.05$, $**P < 0.005$, $***P < 0.001$, $****P < 0.0001$.

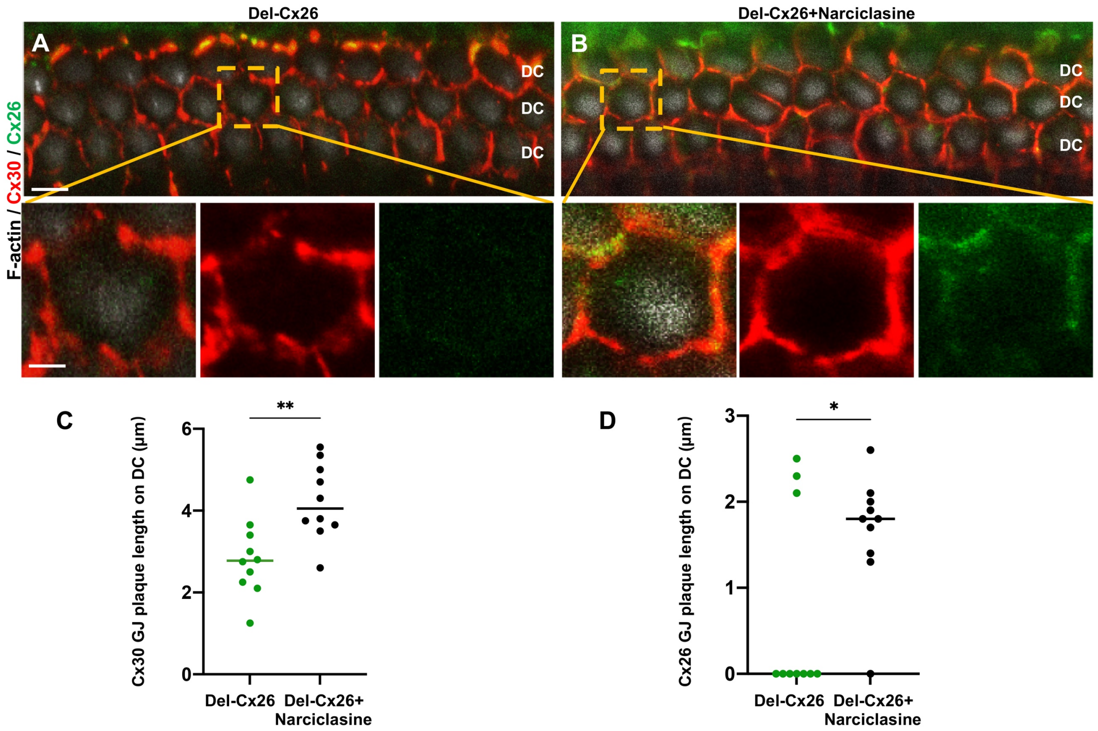

Figure S2 Narciclasine treatment increases membrane localization of Cx30 and Cx26 in DCs in Del-Cx26 mice. (A-B) Immunofluorescence staining of Cx30 (Red), Cx26 (Green), and F-actin (White) in the Del-Cx26 mice (A) and in the Del-Cx26 mice treated with Narciclasine (B). **(C-D)** Quantification of the lengths of Cx30 GJPs (C) and Cx26 GJPs (D) from the Del-Cx26 mice and the Del-Cx26 mice treated with Narciclasine on DCs at P30. Scale bars: 20 μm (panels A-B), and 7 μm (partial enlargement in panels A-B).*P < 0.05, **P < 0.005.

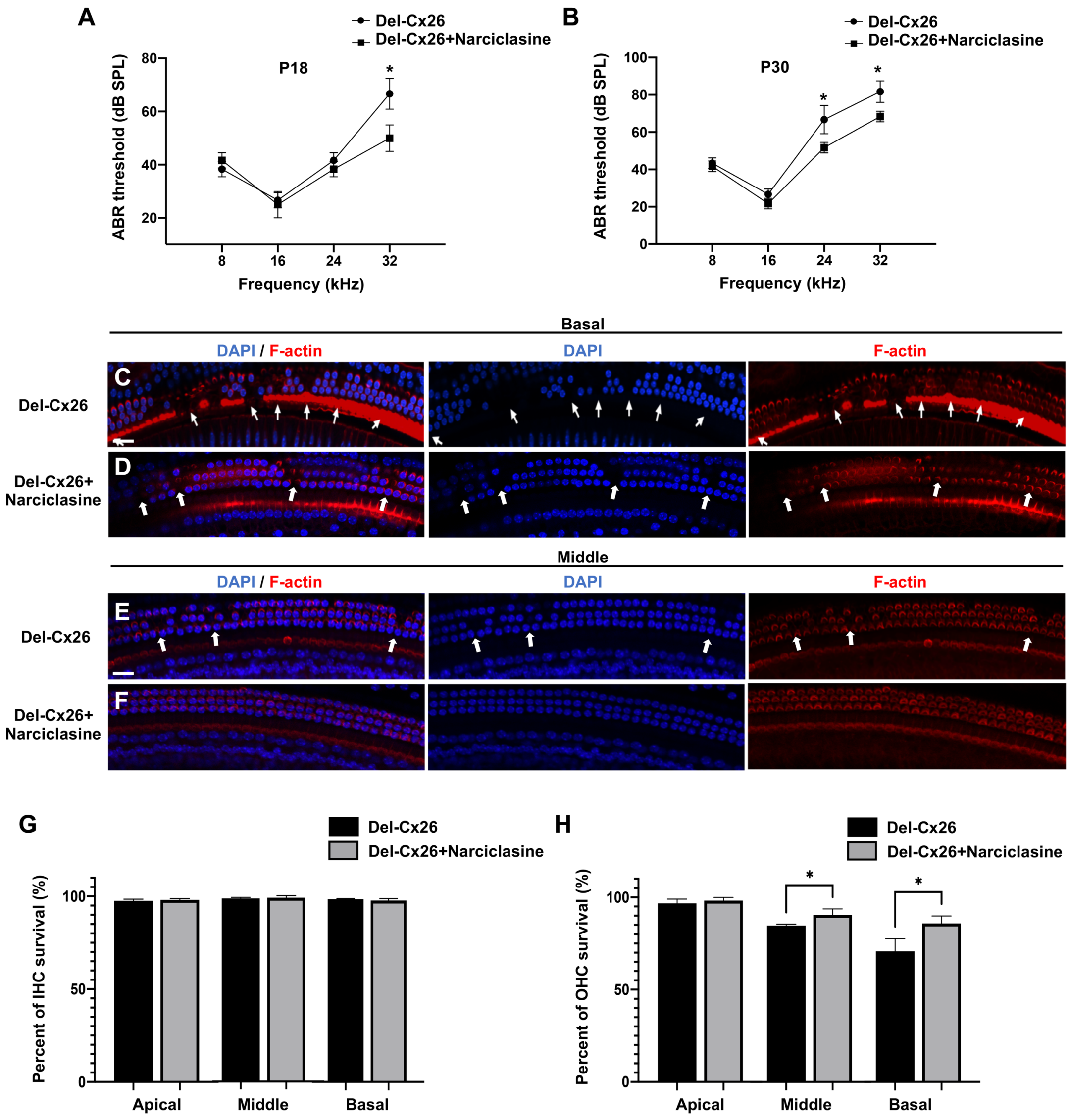

Figure S3 Intraperitoneal injection of Narciclasine rescues hearing loss and hair cell degeneration in Del-Cx26 mice. (A-B) Changes in ABR thresholds at different frequencies in the Del-Cx26 mice and in the Del-Cx26 mice treated with Narciclasine at P18 (A) and P30 (B) (*N*=5). **(C-F)** Representative images of IHCs and OHCs of different turns in the Del-Cx26 mice (C, E) and in the Del-Cx26 mice treated with Narciclasine (D, F) at P30. **(G-H)** Percent of IHCs (G) and OHCs (H) survival in the Del-Cx26 mice and in the Del-Cx26 mice treated with Narciclasine. Scale bars: 20 μm (panels C-F). *P < 0.05.
